# Type 2 diabetes mellitus and cancer: A systematic review and meta-analysis of Mendelian randomization studies

**DOI:** 10.3389/fendo.2026.1713815

**Published:** 2026-04-15

**Authors:** Wei Wu, Guo-liang Huang, Jia Cui

**Affiliations:** 1 Department of Geriatrics, Chun’an Country First People’s Hospital, Chun’an Branch of Zhejiang Provincial People’s Hospital, Hangzhou, China; 2 Department of Nephrology, The Second People’s Hospital of Yuhang District, Hangzhou, China; 3 Department of Endocrinology, Chun’an Country First People’s Hospital, Chun’an Branch of Zhejiang Provincial People’s Hospital, Hangzhou, China

**Keywords:** cancer, Mendelian randomization, meta-analysis, systematic review, type 2 diabetes mellitus

## Abstract

**Background:**

Type 2 diabetes mellitus (T2DM) and cancer are both major global public health concerns; however, their causal relationship remains unclear. This study aims to quantitatively investigate the potential causal associations between T2DM and 17 site-specific cancers through a systematic review and meta-analysis of Mendelian randomization (MR) studies.

**Methods:**

We systematically searched Scopus, PubMed, the Cochrane Library, Web of Science, Embase, and Ovid MEDLINE to identify MR studies investigating the association between T2DM and cancer published up to June 2025. A meta-analysis was performed on extracted data, accompanied by heterogeneity testing, sensitivity analysis, and publication bias assessment.

**Results:**

The initial search yielded 1,143 articles. After multi-level screening, 44 articles were ultimately included, with 42 articles (comprising 131 MR studies) eligible for meta-analysis. The pooled results demonstrated that T2DM was significantly associated with an increased risk of pancreatic cancer (OR = 1.09, 95% CI: 1.04-1.15, *P* = 0.0007) and endometrial cancer (OR = 1.07, 95% CI: 1.04-1.09, *P* < 0.00001). Conversely, T2DM was significantly associated with a decreased risk of gastric cancer (OR = 0.90, 95% CI: 0.85-0.93, *P* < 0.00001), melanoma (OR = 0.97, 95% CI: 0.95-0.99, *P* = 0.009), and esophageal cancer (OR = 0.86, 95% CI: 0.79-0.93, *P* = 0.0002). The effect sizes for T2DM’s associations with thyroid and breast cancers were modest, with no clinical significance. No significant causal association was identified between T2DM and the remaining ten cancer types.

**Conclusion:**

The causal relationship between T2DM and cancer appears to be tissue-specific. T2DM significantly increases the risk of pancreatic and endometrial cancers while demonstrating a negative association with gastric cancer, melanoma, and esophageal cancer.

**Clinical trial registration:**

https://www.crd.york.ac.uk/PROSPERO/, identifier CRD420251066404.

## 1 Introduction

Type 2 diabetes mellitus (T2DM) is a chronic metabolic disease characterized by insulin resistance and β-cell dysfunction, driven by a combination of obesity, sedentary lifestyle, and genetic factors (1, 2). The prevalence of T2DM continues to rise globally, establishing it as a major public health threat (3). T2DM does not exist as an isolated metabolic abnormality. Beyond its well-established association with classic complications such as cardiovascular disease and chronic kidney disease, T2DM may also directly or indirectly modulate cancer initiation and progression (4, 5). These effects are mediated through various pathophysiological pathways, including hyperglycemia, hyperinsulinemia, dysregulation of adipokine secretion, and chronic inflammation (6).

Cancer is a leading cause of premature death worldwide and substantially reduces life expectancy (7). As research has advanced, the association between T2DM and cancer has garnered increasing academic attention (8). However, existing studies are marked by significant limitations and have yet to yield definitive and consistent conclusions. It should be noted that we are not the first to discuss this association; numerous previous observational studies have suggested that T2DM is associated with an elevated risk of several cancers. Nevertheless, these studies have generally been constrained by residual confounding from obesity, dietary patterns, and use of antidiabetic drugs, as well as the potential for reverse causality, rendering their findings less conclusive (9). Therefore, conducting high-quality research to address the limitations of existing studies and to investigate the causal relationship between T2DM and cancer is both necessary and of substantial practical significance.

Mendelian randomization (MR) is an epidemiological method that employs genetic variants as instrumental variables (IVs) to infer causal relationships between exposures and outcomes (10). This approach can effectively overcome the confounding bias and reverse causation that often affect traditional observational studies, thereby offering a novel avenue for exploring the causal link between T2DM and cancer. By utilizing genetic variants that reflect lifelong genetic susceptibility to T2DM, estimates derived from MR studies are more likely to approximate true long-term exposure effects. In recent years, several MR studies have investigated the association between T2DM and individual or a limited number of cancers. However, these studies have been hampered by limitations such as small sample sizes, incomplete coverage of cancer types, and inconsistent findings. Therefore, the present study conducts a systematic review and meta-analysis of published MR studies that meet predefined inclusion criteria. The objective is to quantitatively synthesize the causal effects of genetic susceptibility to T2DM on the risk of 17 site-specific cancers, thereby providing more robust evidence to inform the understanding of this relationship and to guide clinical decision-making.

## 2 Materials and methods

This systematic review was conducted in strict accordance with the Preferred Reporting Items for Systematic Reviews and Meta-Analyses (PRISMA) guidelines (11). The study protocol was prospectively registered on the PROSPERO platform under registration number CRD420251066404. As the study utilized publicly available data, it was exempt from ethical approval and informed consent.

### 2.1 Data sources and search strategy

We systematically searched Scopus, PubMed, the Cochrane Library, Web of Science, Embase, and Ovid MEDLINE to identify MR studies investigating the association between type 2 diabetes and cancer published from the inception of each database to June 11, 2025. The search strategy combined Medical Subject Headings and free-text terms. The complete search strategy is provided in **Supplementary Tables 2**-**S7**.

### 2.2 Data extraction

The initial search yielded 1,143 records, which were imported into EndNote (version 21) for deduplication. Two reviewers (W.W. and J.C.) independently screened titles and abstracts, retaining studies that met the inclusion criteria. Any disagreements were resolved by a third reviewer (G.-L.H.). Following screening, 119 articles underwent full-text assessment, of which 44 met the inclusion criteria and were included in the meta-analysis. For articles reporting multiple outcomes, each exposure-outcome association was treated as an individual study. Where multiple studies examined the same outcome with different sources of exposure or outcome data, all such studies were included.

The inclusion criteria were as follows: (1) T2DM exposure; (2) MR analysis; (3) cancer outcome; (4) reported odds ratio (OR) with 95% confidence interval (CI); and (5) published in English. The exclusion criteria were as follows: (1) non-human studies; (2) non-MR studies; (3) duplicate publications; (4) non-original research, randomized controlled trials, case reports, reviews, commentaries, or conference abstracts; (5) non-English publications (owing to the authors’ language restrictions, this may have introduced language bias); and (6) not reporting the inverse-variance weighted (IVW) OR and its 95% CI.

This study utilized a predesigned data extraction form to ensure systematic and standardized data collection. The information extracted included the first author, ethnicity, year of publication, sample size, outcome measures, and OR with corresponding 95% CI derived from the IVW method. Data were extracted by one author (J.C.) and independently verified by another author (W.W.). In addition, we extracted the number of single-nucleotide polymorphisms (SNPs) used as IVs, the strength of the IVs (F-statistics), the linkage disequilibrium threshold used for IVs selection, and the results of the MR-Egger intercept test, Cochran’s Q test, and MR-PRESSO.

### 2.3 Quality assessment

Two authors (WW and JC) assessed the quality of the included studies using the Strengthening the Reporting of Observational Studies in Epidemiology Using Mendelian Randomization (STROBE-MR) guidelines (12). The guidelines comprised 20 core items. Original quality scores were converted to a percentage scale, and studies were classified into three risk of bias categories: high risk (<75%), moderate risk (75-85%), and low risk (>85%) (13). Disagreements during the assessment were resolved through consensus discussions to ensure the robustness of the meta-analysis results.

### 2.4 Statistical analysis

A meta-analysis was conducted when two or more independent studies examined the causal relationship between T2DM and a specific cancer. The primary effect measure was the OR with its 95% CI, used to assess the strength of the association between T2DM and cancer risk. Cochran’s Q test and the I²statistic were employed to evaluate heterogeneity among studies. The I² value represents the percentage of total variation across studies attributable to heterogeneity, interpreted as follows: 25%-50% indicated low heterogeneity, 50%-75% indicated moderate heterogeneity, and >75% indicated high heterogeneity. Heterogeneity was considered present when I² exceeded 50% and the *P*-value from Cochran’s Q test was less than 0.05, prompting the use of a random-effects model (**Table 1**). When the number of included studies was more than three, the leave-one-out sensitivity analysis was further performed to evaluate the influence of individual studies on the pooled results. Publication bias was evaluated using funnel plots, Begg’s test, and Egger’s test. If bias was detected, the trim-and-fill method was applied for correction. Data analysis for this study was conducted using RevMan (version 5.4), Stata (versions 15.0 and 18.0), and RStudio (version 4.4.2). *P*-value < 0.05 was considered statistically significant.

**Table 1:** Begg’s test, Egger’s test, and Cochran’s Q test.

	Begg’s test (adjusted Kendall’s score)			Egger’s test (bias coefficient)			Cochran’s Q test		
Cancer type	*z* score	*P* value	*P* value (continuity corrected)	Coefficient	*t* value	*P* value	Chi²	df	*P* value
Bladder cancer	0.52	0.602	1.000	1.8058	1.69	0.340	8.60	2	0.01
Breast cancer	-1.62	0.105	0.112	-0.8411	-1.18	0.255	25.52	19	0.14
Colorectal cancer	-1.97	0.049	0.055	-3.3524	-1.74	0.108	91.83	13	<0.00001
Endometrial cancer	-1.52	0.129	0.172	-2.9407	-3.46	0.018	7.06	6	0.31
Esophageal cancer	-0.49	0.624	0.806	-2.7025	-0.73	0.517	8.05	4	0.09
Gastric cancer	2.26	0.024	0.029	2.0532	2.33	0.045	19.01	10	0.04
Glioblastoma	2.23	0.026	0.035	3.1014	3.83	0.009	17.90	7	0.01
Liver cancer	2.95	0.003	0.004	4.6984	2.81	0.023	50.28	9	<0.00001
Lung cancer	-0.45	0.652	0.764	-0.4581	-0.44	0.675	12.22	6	0.06
Lymphoma	-1.96	0.050	0.086	-0.5718	-0.92	0.424	1.24	4	0.87
Melanoma	-0.54	0.586	0.640	-1.3981	-2.57	0.030	45.07	10	<0.00001
Ovary cancer	-0.49	0.621	0.711	0.582	0.72	0.496	7.17	7	0.41
Pancreatic cancer	-0.60	0.547	0.584	1.6729	1.99	0.070	51.21	13	<0.00001
All cancer	0.33	0.742	0.773	0.1397	0.13	0.896	118.12	16	<0.00001

df, degrees of freedom.

## 3 Results

### 3.1 Study selection

**Figure 1:**
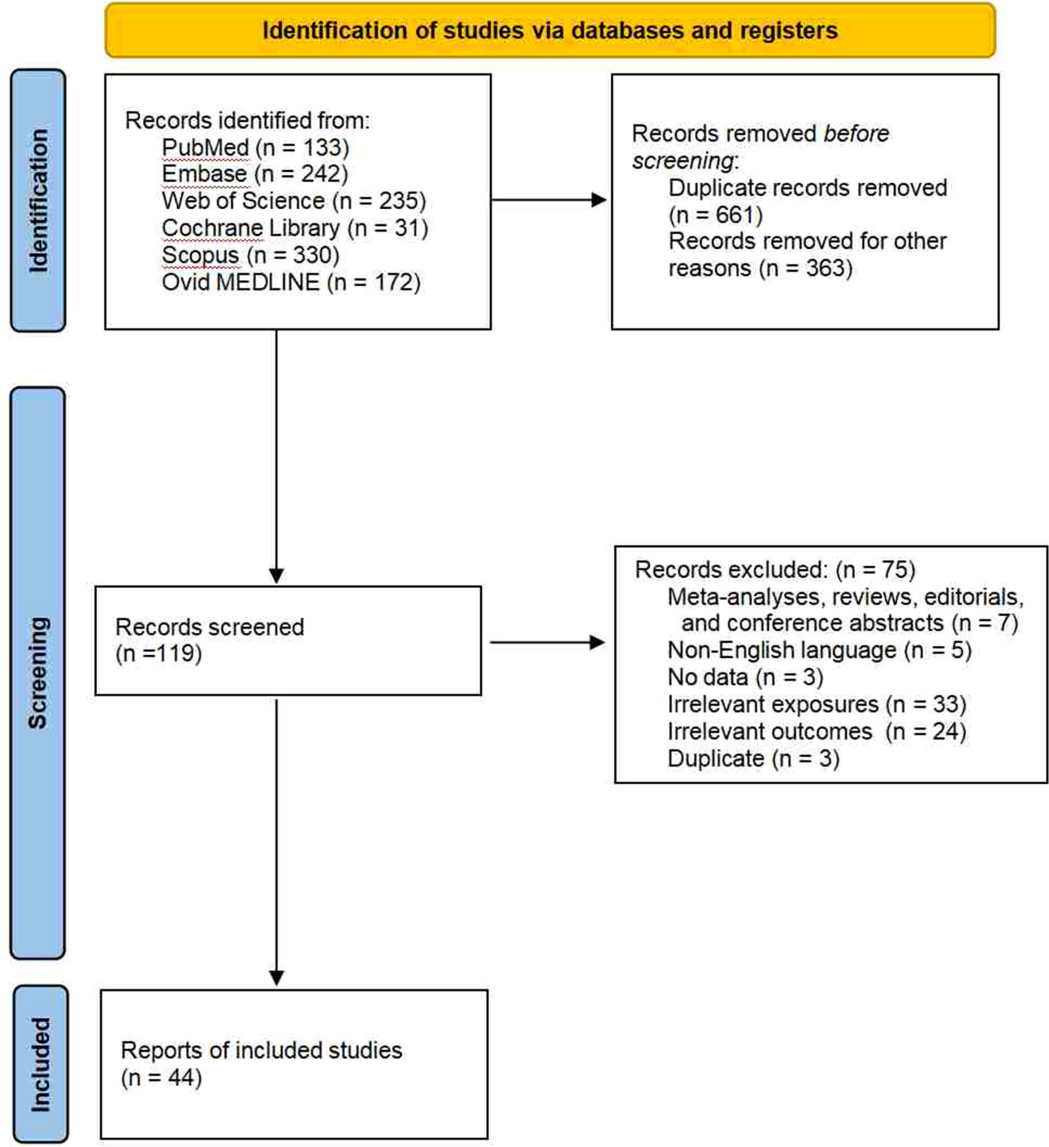
Preferred reporting items for systematic reviews and meta-analysis flow diagram.

The initial search identified 1,143 records. After removing 661 duplicate records using EndNote software and manual checks, 363 records were excluded for other reasons (e.g., publication type, language), leaving 119 articles for full-text review. Following rigorous assessment, 44 articles met the inclusion criteria (**Figure 1**). It should be noted that only one relevant study on gallbladder cancer was ultimately identified; therefore, a meta-analysis could not be performed (14). Another study by Lin et al. was excluded from the final analysis because of an excessively narrow confidence interval (OR = 0.9996; 95% CI: 0.9995-0.9998) (15). A total of 131 Mendelian randomization study results were included.

### 3.2 Baseline characteristics

The included studies were published between 2018 and 2025. The number of single nucleotide polymorphisms (SNPs) used ranged from 10 to 497, with two studies not reporting the exact number (16, 17). Regarding the strength of IVs, the F-statistics in the included studies generally exceeded 10, satisfying the criterion for strong IVs. The linkage disequilibrium threshold was commonly set at r²< 0.001 with a window size of 10,000 kb to avoid confounding by linkage disequilibrium. The study populations were predominantly of European ancestry but also included East Asian, North American, and South American populations. Seventeen cancer types were investigated: bladder cancer (18–20), prostate cancer (20, 21), colorectal cancer (20, 22–27), pancreatic cancer (20, 22, 23, 25, 28–32), gastric cancer (20, 22, 23, 25, 33–35), liver cancer (20, 22, 23, 36–38), esophageal cancer (20, 22, 23), lung cancer (17, 20, 25, 39, 40), kidney cancer (20, 41), thyroid cancer (20, 42), ovarian cancer (20, 43), endometrial cancer (20, 27, 44, 45), breast cancer (16, 20, 21, 46, 47), glioblastoma (48–51), melanoma (20, 52–54), oral cancer (55, 56), and lymphoma (20, 57). All included studies used the IVW method as their primary analytical approach. Baseline characteristics of the included studies are summarized in **Table 2**.

**Table 2:** Characteristics of included studies.

Author	Year	Ethnicity	*P*	F	LD thresholds	Exposure sample size	Outcome (sample size)
An et al. (22)	2024	East Asian	< 5×10^-6^	>10	r^2^ < 0.001, ws = 10,000kb	40,250 cases; 170,615 controls; 36,614 cases; 155,150 controls; 77,418 cases; 356,122controls	Colorectal cancer (7,062 cases; 195,745 controls); Esophageal cancer (4,050 cases; 208,403controls); Gastric cancer (6,563 cases; 195,745 controls); Hepatocellular carcinoma (1,866 cases; 195,745 controls); Pancreatic cancer (442 cases; 195,745 controls);
Chen et al. (23)	2023	European	< 5×10^-8^	NA	r^2^ < 0.001, ws = 10,000kb	148,726 cases; 965,732 controls	Colorectal cancer (9,519 cases; 686,953 controls); Esophageal cancer (1,130 cases; 702,116 controls); Gastric cancer (1,608 cases; 701,472 controls); Pancreatic cancer (5,478 cases; 1,223,335controls); Liver cancer (862 cases; 702,008 controls)
Chen et al. (48)	2024	European	< 5×10^-6^	NA	r^2^ < 0.001, ws = 5000kb	61,714 cases; 117,8 controls	Glioblastoma (91 cases; 218,701 controls)
Chen et al. (17)	2024	East Asian	< 5×10^-8^	>10	r^2^ < 0.001, ws = 10,000kb	40,250 cases; 170,615 controls	Lung cancer (212,453 cases; 208,403 controls)
Cheng et al. (14)	2024	European; East Asian	< 5×10^-8^	>10	r^2^ < 0.01, ws = 10,000kb	61,714 cases; 1,178 controls	Gallbladder cancer (41 cases; 866 controls)
Gormley et al. (55)	2022	European; North America; South America	< 5×10^-8^	>10	r^2^ < 0.001	228,499 cases; 1,178,783 controls	Oral and oropharyngeal cancer combined (6,034 cases; 6,585 controls)
He et al. (33)	2025	East Asian	< 5×10^-8^	>10	r^2^ < 0.001, ws = 10,000kb	40,250 cases; 170,615 controls	Gastric cancer (6,563 cases; 195,745 controls)
He et al. (18)	2025	European	< 5×10^-8^	>10	r^2^ < 0.001, ws = 10,000kb	655,666 sample size	Bladder cancer (373,295 sample size)
Hong et al. (39)	2021	Asian	< 5×10^-8^	NA	r^2^ < 0.10	NA	Lung cancer (13,327 cases; 13,328 controls)
Huang et al. (42)	2022	European	< 5×10^-8^	>10	r^2^ < 0.01	NA	Thyroid cancer (989 cases; 217,803 controls)
Huang et al. (56)	2024	European	< 5×10^-8^	NA	r^2^ < 0.001, ws = 10,000kb	32,469 cases; 183,185 controls	Oropharyngeal cancer (1,119 cases; 2,329 controls)
Huo et al. (36)	2024	East Asian	< 5×10^-8^	NA	r^2^ < 0.001, ws = 10,000kb	77,418 cases; 356,122 controls	Hepatocellular carcinoma (1,866 cases; 195,745 controls)
Johansson et al. (41)	2019	European	< 5×10^-8^	NA	r^2^ < 0.1	NA	Renal cell carcinoma (10,784 patients; 20,406 controls)
Ke et al. (28)	2024	European	< 5×10^-8^	>10	r^2^ < 0.2, ws = 250kb	NA	Pancreatic cancer: FinnGen (NA); Pancreatic cancer: FinnGen+UKBB (NA); Pancreatic cancer: UKBB (NA)
Ke et al. (24)	2024	European	< 5×10^-8^	>10	r^2^ < 0.001, ws = 10,000kb	298,957 sample size; 655,666 sample size	Colorectal cancer (32,072 sample size)
Larsson et al. (19)	2025	European	< 5×10^-8^	>30	r^2^ < 0.01	228,499 cases; 1,178,783 controls	Bladder cancer (6,984 cases; 708,432 controls)
Li et al. (50)	2024	European	< 5×10^-8^	>10	r^2^ < 0.001, ws = 10,000kb	61,714 cases; 1,178 controls	Cranial neuroma (357 cases; 218,701 controls); Malignant meningioma (640 cases; 218,701 controls); Pituitary and craniopharyngioma (735 cases; 218,701 controls); Spinal cord tumors (196 cases; 218,596 controls); Glioblastoma (91 cases; 218,701 controls)
Li et al. (25)	2025	European	< 5×10^-6^	>10	r^2^ < 0.001, ws = 10,000kb	298,957 sample size	Colorectum neuroendocrine neoplasms (314,544 sample size); Lung neuroendocrine neoplasm (314,370 sample size); Pancreas neuroendocrine neoplasms (314,322 sample size); Stomach neuroendocrine neoplasms (314,311 sample size)
Li et al. (49)	2025	European	< 1×10^-5^	NA	r^2^ < 0.01, ws = 10,000kb	243 cases; 287,137 controls	Glioblastoma multiforme (243 cases; 287,137 controls)
Lin et al. (15)	2025	European	< 5×10^-8^	NA	r^2^ < 0.001, ws = 10,000kb	12,931 cases; 57,196 controls; 61,714 cases; 1,178 controls; 48,286 cases; 250,671 controls; 32,469 cases;183,185 controls; 29,166 cases; 183,185 controls; 29,193 cases; 182,573 controls; 17,268 cases; 184,778 controls;	Esophageal cancer (740 cases; 372,016 controls)
Liu et al. (51)	2023	European	< 5×10^-8^	>10	r^2^ < 0.001, ws = 10,000kb	61,714 cases; 1,178 controls	Glioma (1,856 cases; 4,955 controls)
Lu et al. (29)	2020	European	< 1×10^-6^	NA	r^2^ < 0.001, ws = 10,000kb	62,892 cases; 596,424 controls	Pancreatic ductal adenocarcinoma (8,769 cases; 7,055 controls)
Ma et al. (34)	2025	East Asian; European	< 5×10^-8^	NA	r^2^ < 0.001, ws = 10,000kb	36,614 cases; 155,150 controls: 38,841 cases; 451,248 controls	Gastric cancer (1,029 cases; 475,087 controls)
Molina-Montes et al. (30)	2020	European	≤ 5 ×10^-5^	NA	MAF ≥ 0.05	736 cases; 2,822 controls	Pancreatic cancer (2,018 cases; 1,540 controls)
Murphy et al. (26)	2022	European	< 5×10^-8^	>516	r^2^ < 0.01, ws = 10,000kb	74,124 cases; 824,006 controls	Colon cancer (48,214 cases; 64,159 controls); Colorectal cancer (48,214 cases; 64,159 controls); Distal colon cancer (48,214 cases; 64,159 controls); Proximal colon cancer (48,214 cases; 64,159 controls); Rectal cancer (48,214 cases; 64,159 controls)
Nead et al. (44)	2015	European	< 5×10^-8^	NA	MAF ≥ 0.05, call rate>95%; MAF < 0.05, call rate>99%	NA	Endometrial cancer (1,287 cases; 8,273 controls)
Qian et al. (52)	2024	European	< 5×10^-8^	>10	r^2^ < 0.001, ws = 10,000kb	80,154 cases; 853,816 controls	Skin cancer-basal cell carcinoma (20,791 cases; 286,893 controls); Skin cancer-basal cell carcinoma (18,982 cases; 287,137 controls); Skin cancer-melanoma (30,134 cases; 81,415 controls); Skin cancer-melanoma (3,960 cases; 286,874 controls); Skin cancer-squamous cell carcinoma (7,402 cases; 286,892 controls); Skin cancer-squamous cell carcinoma (3,251 cases; 287,137 controls);
Shen et al. (31)	2023	East Asian	< 5×10^-8^	NA	r^2^ < 0.1	428,362 cases	Pancreatic cancer (NA)
Verdiesen et al. (16)	2025	Multiancestry; European;	< 1×10^-8^	>10	r^2^ < 0.001	228,499 cases; 1,178,783 controls	All BCAC breast cancer (133,384 cases; 113,789 controls); HER2-enriched breast cancer (2,884 cases; 91,477 controls); Luminal A-like breast cancer (45,253 cases; 91,477 controls); Luminal B-/HER2-negative-like breast cancer (6,350 cases; 91,477 controls); Luminal B-like breast cancer (6,427 cases; 91,477 controls); Triple-negative breast cancer (8,602 cases; 91,477 controls);
Wang et al. (32)	2025	European	< 1 ×10^-5^	>10	r^2^ < 0.001, ws = 10,000kb	215,654 participants	Pancreatic cancer (476,245 participants)
Wang et al. (35)	2025	East Asian; European	< 5×10^-8^	>10	r^2^ < 0.001, ws = 10,000kb	45,383 cases; 132,032 controls; 32,469 cases; 183,185 controls	Gastric cancer (7,921 cases; 159,201 controls); Gastric cancer (1,029 cases; 475,087 controls)
Wei et al. (37)	2024	East Asian; European	≤ 5× 10^−8^	>10	r^2^ < 0.001, ws = 10,000kb	40,250 cases; 170,615 controls; 62,892 cases; 596,424 controls	Hepatocellular carcinoma (1,866 cases; 95,745 controls); Hepatocellular carcinoma (406 cases; 49,302 controls); Hepatocellular carcinoma (123 cases; 456,225 controls)
Yarmolinsky et al. (43)	2019	European	< 5×10^-8^	≥ 10	r^2^ < 0.001, ws = 10,000kb	12,171 cases; 56,862 controls	Clear cell carcinoma (1,366 cases; 40,941 controls); Endometrioid carcinoma (2,810 cases; 40,941 controls); High grade serous carcinoma (13,037 cases; 40,941 controls); Invasive epithelial ovarian cancer (22,406 cases; 40,941 controls); Low grade serous carcinoma (1,012 cases; 40,941 controls); Low malignant potential tumors (3,103 cases; 40,941 controls); Mucinous carcinoma (1,417 cases; 40,941 controls)
Yeung et al. (21)	2019	European	NA	≥ 10	r^2^ < 0.001	74,124 cases; 824,006 controls	Breast cancer (122,977 cases; 105,974 controls); Prostate cancer (79,148 cases; 61,106 controls)
Yu et al. (40)	2024	European	< 5×10^-8^	>10	r^2^ < 0.001, ws = 10,000kb	26,676 cases; 132,532 controls	Lung adenocarcinoma (1,590 cases; 410,591 controls); Lung squamous cell carcinoma (1,510 cases; 410,671 controls); Small cell lung cancer (717 cases; 411,464 controls)
Yuan et al. (20)	2020	European	< 1 ×10^-5^	NA	NA	74,124 cases; 824,006 controls	Bladder cancer (2,588 cases; 292,606 controls); Breast ER- (BCAC) (21,468 cases; 105,974 controls); Breast ER+ (BCAC) (69,501 cases; 105,974 controls); Breast(BCAC) (122,977 cases; 105,974 controls); Breast(UKBB) (13,666 cases; 353,977 controls); Cervix cancer (1,928 cases; 292,606 controls); Esophagus cancer (843 cases; 292,606 controls); Lung cancer (2,838 cases; 292,606 controls); Melanoma (4,869 cases; 292,606 controls); Non-Hodgkin lymphoma (2,296 cases; 292,606 controls); Ovary cancer (1,520 cases; 292,606 controls); Pancreatic cancer (9,040 cases; 12,496 controls); Prostate cancer (7,872 cases; 292,606 controls); Thyroid Cancer (375 cases; 292,606 controls); Uterus cancer (1,931 cases; 292,606 controls); Colorectum cancer (5,486 cases; 292,606 controls); Kidney cancer (1,310 cases; 292,606 controls) Liver cancer (324 cases; 292,606 controls); Stomach cancer (736 cases; 292,606 controls);
Zhang et al. (53)	2023	European	< 5×10^-8^	>10	r^2^ < 0.001, ws = 10,000kb	74,124 cases; 824,006 controls	Melanoma (UKBB:3,598 cases; 459,335 controls; FinnGen: 393 cases; 180,622 controls)
Zhang et al. (46)	2024	European	< 5×10^-8^	>10	r^2^ < 0.001, ws = 10,000kb	61,714 cases; 1178 controls	Breast cancer (122,977 cases; 105,974 controls); ER- Breast cancer (21,468 cases; 105,974 controls); ER+ Breast cancer (69,501 cases; 105,974 controls)
Zhang et al. (27)	2022	European	< 5×10^-8^	>10	r^2^ < 0.01	228,499 cases; 1,178,783 controls	Colorectal cancer (20,049 cases; 22,661 controls); Endometrial cancer (12,906 cases; 108,979 controls)
Zhang et al. (38)	2024	European	< 5×10^-8^	>10	r^2^ < 0.01, ws = 10kb	228,499 cases; 1,178,783 controls	Liver cancer (NA)
Zhang et al. (45)	2024	European	≤ 5× 10^-8^	>10	r^2^ < 0.01	490,089 sample size	All-Endometrioid adenocarcinoma (9,988 cases; 2,918 controls); Endometrioid adenocarcinoma (8,758 cases; 2,918 controls); Non-endometrioid adenocarcinoma (1,230 cases; 2,918 controls)
Zhao et al. (57)	2025	European	< 5×10^-8^	>10	r^2^ < 0.001, ws = 10,000kb	61,714 cases; 1,178 controls	Diffuse large B-cell lymphoma (1,050 cases; 314,193 controls); Follicular lymphoma (394 cases; 455,954 controls); Lymph node tumor (908 cases; 455,440 controls); Non-Hodgkin’s lymphoma (2,400 cases; 410,350 controls)
Zhao et al. (54)	2024	European	< 5×10^-8^	>10	r^2^ < 0.01, ws = 10,000kb	80,154 cases; 853,816 controls	Basal cell carcinoma (20,506 cases; 314,193 controls); Cutaneous melanoma (3,194 cases; 314,193 controls); Cutaneous squamous cell carcinoma (3,531 cases; 314,193 controls)
Zhao et al. (47)	2025	European	< 5×10^-8^	>10	r^2^ < 0.001, ws = 10,000kb	38,841 cases; 451,248 controls	HER2-Enriched-like (106,278 cases; 91,477 controls); Luminal B/HER2-negative-like (106,278 cases; 91,477 controls); Luminal B-like (106,278 cases; 91,477 controls); Luminal A-like (106,278 cases; 91,477 controls); Overall breast cancer (133,384 cases; 113,789 controls); Triple negative breast cancer (106,278 cases; 91,477 controls)

*P*, Genome-wide significance *P*-value threshold; F, F-statistic; LD, linkage disequilibrium; SNP, single nucleotide polymorphism; NA, not available; ws, window size; MAF, minor allele frequency; UKBB, UK Biobank; BCAC, Breast cancer association consortium; ER, estrogen receptor.

### 3.3 Protective factors

**Figure 2:**
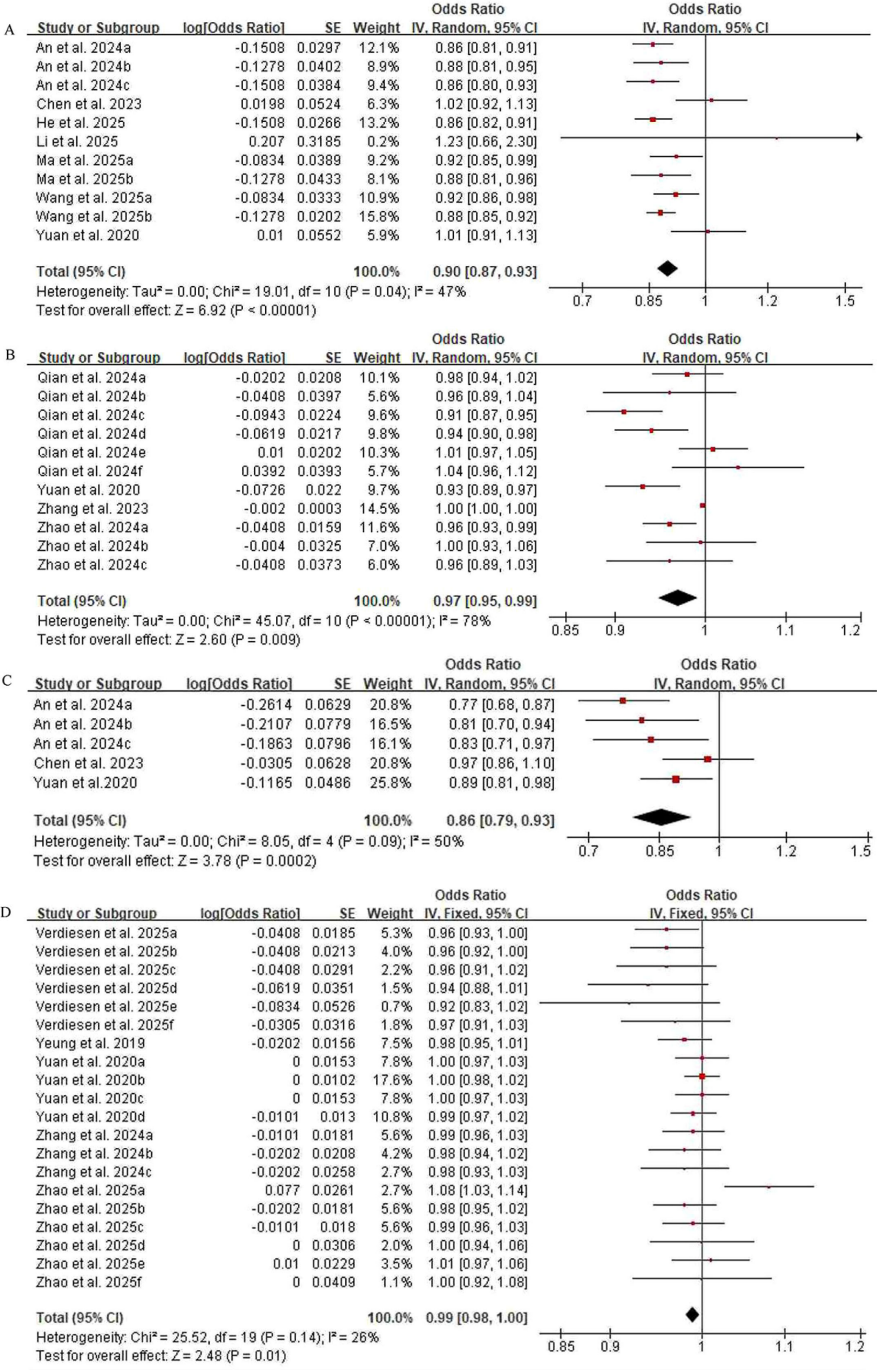
Forest plot of the association between T2DM and reduced cancer risk. **(A)** Gastric cancer, **(B)** Melanoma, **(C)** Esophageal cancer, **(D)** Breast cancer. SE,standard error; IV, inverse variance; CI, confidence interval; T2DM, type 2 diabetes mellitus.

Based on heterogeneity tests, random-effects models were employed to analyze the causal associations of T2DM with gastric cancer, melanoma, and esophageal cancer. The results indicated that T2DM was inversely associated with gastric cancer (OR = 0.90, 95% CI: 0.85-0.93, *P* < 0.00001), melanoma (OR = 0.97, 95% CI: 0.92-0.99, *P* = 0.009), and esophageal cancer (OR = 0.86, 95% CI: 0.74-0.93, *P* = 0.0002), suggesting a potential protective effect of T2DM against these cancers (**Figure 2**).

### 3.4 Risk factors

**Figure 3:**
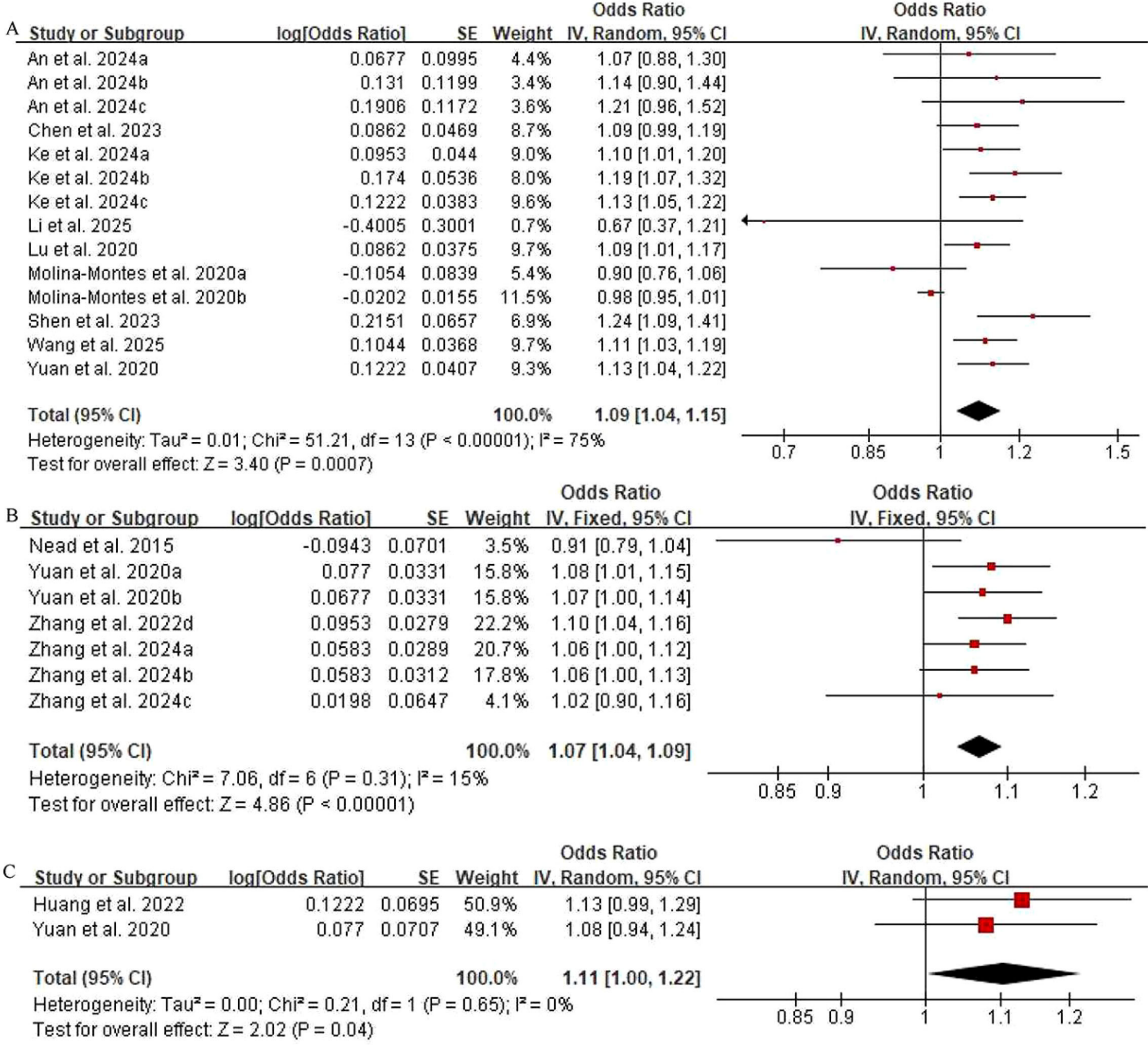
Forest plot of the association between T2DM and increased cancer risk. **(A)** Pancreatic cancer, **(B)** Endometrial cancer, **(C)** Thyroid cancer. SE,standard error; IV, inverse variance; CI, confidence interval; T2DM, type 2 diabetes mellitus.

The results showed that T2DM was associated with an increased risk of pancreatic cancer (OR = 1.09, 95% CI: 1.04-1.15, *P* = 0.0007) and endometrial cancer (OR = 1.07, 95% CI: 1.04-1.09, P < 0.00001). Although thyroid cancer also exhibited a trend toward increased risk (OR = 1.11, 95% CI: 1.00-1.22, *P* = 0.04), the clinical significance of this association remained uncertain, as the CI included the null value and the effect size was modest (**Figure 3**). For pancreatic cancer, despite the presence of high heterogeneity, leave-one-out analysis did not identify any single study exerting a decisive influence on the pooled effect estimate (**Supplementary Figure 3**).

### 3.5 Factors with no significant correlation

**Figure 4:**
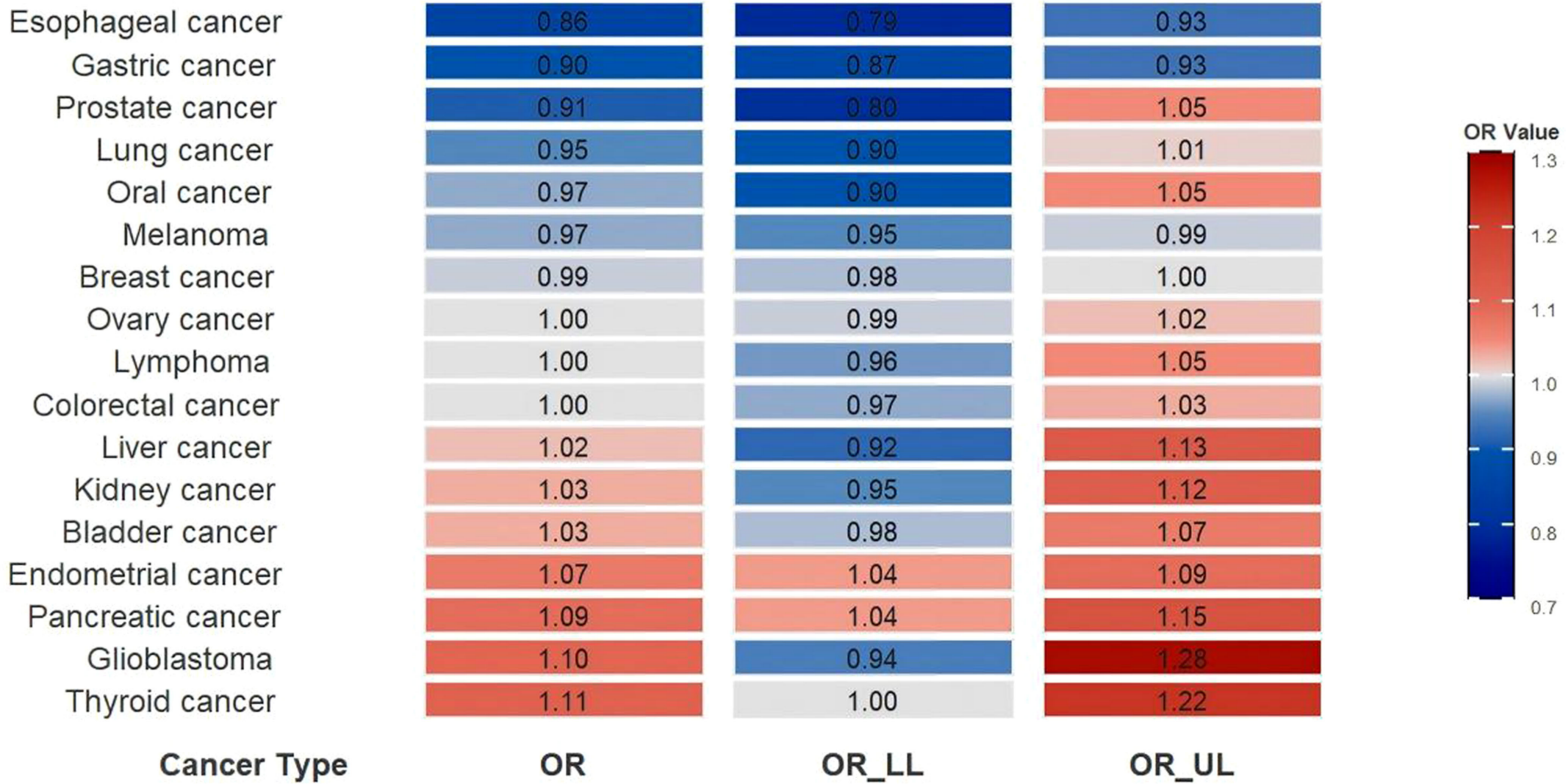
Heatmap of the association between T2DM and cancer risk. OR, odds ratio; OR_LL, odds ratio-lower limit; OR_UL, odds ratio-upper limit.

No statistically significant causal associations were detected between T2DM and bladder cancer, prostate cancer, colorectal cancer, liver cancer, lung cancer, kidney cancer, glioblastoma, ovarian cancer, oral cancer, or lymphoma (**Figure 4**). Heterogeneity analysis indicated no significant heterogeneity among studies on lymphoma or ovarian cancer; therefore, fixed-effects models were employed (**Table 1**). Random-effects models were used for the remaining cancer types.

### 3.6 Sensitivity analysis and publication bias

Leave-one-out sensitivity analysis showed that the direction of the pooled effect size for the aforementioned associations did not change after the sequential exclusion of any single study, suggesting robust results (**Supplementary Figures 1**-**S4**). Funnel plots, Begger’s test and Egger’s test suggested potential publication bias among studies on gastric cancer, liver cancer, glioblastoma, melanoma, and endometrial cancer (**Table 1**; **Supplementary Figures 5**-**S9**). After applying the trim-and-fill method for correction, the direction of the pooled results remained unchanged, indicating that the overall findings of this study were robust (**Supplementary Figures 5**-**S9**).

### 3.7 Evaluation of study quality

The quality of the included studies was assessed using the STROBE-MR guideline. The results indicated that the studies generally provided comprehensive methodological and outcome information and were rated as having a low risk of bias. Overall, the methodological quality of the included studies was high, and the risk of bias was within an acceptable range, supporting the reliability of the study’s conclusions (**Table 3**).

**Table 3:** Quality assessment based on STROBE-MR guidelines.

Author	Abstract	Introduction		Methods						Results				Discussion				Other information				
	Title and abstract	Back- ground	Objectives	Study design and data sources	Assumptions	Statistical methods main analysis	Assessment of assumptions	Sensitivity and additional analysis	Software and preregistration	Descriptive data	Main results	Assessment of assumptions	Sensitivity and additional analysis	Key results	Limitations	Interpretation	Generalizability	Funding	Data and data sharing	Conflicts of interest	Total score (out of 20)	Score (%)
An et al. (22)	1	1	1	1	1	1	1	1	1	1	1	1	1	1	1	1	1	0.5	1	1	19.5	97.5
Chen et al. (23)	1	1	1	1	1	1	1	0.5	1	1	1	1	0.5	1	1	1	1	1	1	0	18	90
Chen et al. (48)	1	1	1	1	1	1	1	1	1	1	1	0.5	1	1	1	1	1	1	1	1	19.5	97.5
Chen et al. (17)	1	1	1	1	1	0.5	1	1	0.5	1	1	1	1	1	1	1	1	0.5	1	1	18.5	92.5
Cheng et al. (14)	1	1	1	1	1	1	1	1	1	1	1	1	1	1	1	1	1	1	1	1	20	100
Gormley et al. (55)	1	1	1	1	1	1	1	1	1	1	1	1	0.5	1	1	1	1	1	1	1	19.5	97.5
He et al. (33)	1	1	1	1	1	1	1	1	1	1	1	1	1	1	1	1	1	0.5	1	1	19.5	97.5
He et al. (18)	1	1	1	1	1	1	1	0.5	1	1	1	1	0.5	1	1	1	1	0.5	1	1	18.5	92.5
Hong et al. (39)	1	1	1	0.5	1	1	0.5	1	1	1	1	1	1	1	1	1	1	1	0.5	1	18.5	92.5
Huang et al. (42)	1	1	1	0.5	1	1	1	1	1	1	1	1	0.5	1	1	1	1	1	1	1	19	95
Huang et al. (56)	1	1	1	1	1	1	1	1	1	1	1	1	1	1	0.5	1	1	1	1	1	19.5	97.5
Huo et al. (36)	1	1	1	1	1	1	1	1	1	1	1	1	1	1	1	1	1	1	1	1	20	100
Johansson et al. (41)	1	1	1	0.5	1	1	1	1	1	1	1	1	1	1	0	1	1	1	0.5	0.5	18.5	92.5
Ke et al. (28)	1	1	1	0.5	1	1	1	1	1	1	1	1	1	1	1	1	1	1	0.5	1	19	95
Ke et al. (24)	1	1	1	1	1	1	1	1	1	1	1	1	1	1	1	1	1	1	1	1	20	100
Larsson et al. (19)	1	1	1	1	1	1	1	1	0.5	1	1	1	0.5	1	1	1	1	1	1	1	19	95
Li et al. (50)	1	1	1	1	1	1	1	1	1	1	1	1	0.5	1	1	1	1	0.5	1	1	19	95
Li et al. (25)	1	1	1	1	1	1	1	1	1	1	1	1	1	1	1	1	1	0.5	1	1	19.5	97.5
Li et al. (49)	1	1	1	1	1	1	1	1	1	1	1	1	0.5	1	0.5	1	1	0.5	1	1	18.5	92.5
Lin et al. (15)	1	1	1	1	1	1	1	1	1	1	0.5	1	0.5	1	1	1	1	1	1	1	19	95
Liu et al. (51)	1	1	1	1	1	1	1	1	0.5	1	1	1	0.5	1	1	1	1	1	1	1	19	95
Lu et al. (29)	1	1	1	1	1	1	1	1	1	1	1	1	1	1	0.5	1	1	1	1	1	19.5	97.5
Ma et al. (34)	1	1	1	1	1	1	1	1	1	1	1	1	1	1	1	1	1	1	1	1	20	100
Molina-Montes et al. (30)	1	1	1	1	1	1	1	1	1	1	1	1	0.5	1	1	1	1	1	1	1	19.5	97.5
Murphy et al. (26)	1	1	1	1	1	1	1	1	0.5	1	1	1	1	1	1	1	1	1	1	1	19.5	97.5
Nead et al. (44)	1	1	1	1	1	1	1	0.5	1	1	1	1	0.5	1	1	1	1	1	0.5	0.5	18	90
Qian et al. (52)	1	1	1	1	1	1	1	1	1	1	1	1	0.5	1	1	1	1	1	1	1	19.5	97.5
Shen et al. (31)	1	1	1	1	1	1	1	0.5	1	1	1	1	0.5	1	1	1	1	1	1	1	19	95
Verdiesen et al. (16)	1	1	1	1	1	1	1	1	1	1	1	1	1	1	0.5	1	1	1	1	0.5	19	95
Wang et al. (32)	1	1	1	1	1	1	1	1	1	1	1	1	1	1	1	1	1	1	1	1	20	100
Wang et al. (35)	1	1	1	1	1	1	1	1	1	1	1	1	1	1	1	1	1	1	1	1	20	100
Wei et al. (37)	1	1	1	1	1	1	1	1	1	1	1	1	1	1	1	1	1	1	1	1	20	100
Yarmolinsky et al. (43)	1	1	1	1	1	1	1	1	1	1	1	1	1	1	1	1	1	1	1	0.5	19.5	97.5
Yeung et al. (21)	1	1	1	1	1	1	1	1	1	1	1	1	1	1	1	1	1	1	1	1	20	100
Yu et al. (40)	1	1	1	1	1	1	1	1	1	1	1	1	0.5	1	1	1	1	1	1	1	19.5	97.5
Yuan et al. (20)	1	1	1	1	1	1	1	1	1	1	1	1	0.5	1	0.5	1	1	1	1	1	19	95
Zhang et al. (53)	1	1	1	1	1	1	1	1	0.5	1	1	1	1	1	1	1	1	1	1	1	19.5	97.5
Zhang et al. (46)	1	1	1	1	1	1	1	1	1	1	1	1	1	1	1	1	1	1	1	1	20	100
Zhang et al. (27)	1	1	1	1	1	1	1	1	1	1	1	1	1	1	1	1	1	0	1	1	19	95
Zhang et al. (38)	1	1	1	0.5	1	1	1	1	1	1	1	1	1	1	1	1	1	0	1	1	19	95
Zhang et al. (45)	1	1	1	1	1	1	1	1	1	1	1	1	1	1	1	1	1	0	1	1	19	95
Zhao et al. (57)	1	1	1	1	1	1	1	1	1	1	1	1	1	1	1	1	1	1	1	1	20	100
Zhao et al. (54)	1	1	1	1	1	1	1	1	1	1	1	1	1	1	1	1	1	1	1	1	20	100
Zhao et al. (47)	1	1	1	1	1	1	1	1	0.5	1	1	1	1	1	1	1	1	1	1	1	19.5	97.5

## 4 Discussion

This study systematically evaluated the causal association between T2DM and 17 cancer types using a meta-analysis of 131 MR studies. The results demonstrated that T2DM increased the risk of pancreatic cancer and endometrial cancer, while exhibiting inverse associations with gastric cancer, melanoma, and esophageal cancer. The statistical findings for thyroid cancer and breast cancer, owing to their minimal effect sizes, were deemed clinically insignificant and were not considered indicative of causal relationships. Furthermore, no significant associations were observed between T2DM and the remaining ten cancer types. These findings suggest that T2DM may exert distinct etiological roles across different cancer types, highlighting its tissue-specific effects in carcinogenesis.

A positive association between T2DM and pancreatic cancer risk was observed in this study, consistent with epidemiological evidence. A retrospective cohort study by Yau et al., which included 197,906 patients from the Hong Kong electronic health database, reported a significantly higher incidence of pancreatic cancer in patients with T2DM compared to nondiabetic individuals (58). Mechanistically, persistent hyperglycemia promotes the accumulation of advanced glycation end-products in pancreatic tissue, increases digestive enzyme secretion, and triggers inflammatory responses, ultimately facilitating tumorigenesis (59). Additionally, pancreatic stellate cells, which are widely recognized as the primary precursors of cancer-associated fibroblasts, can induce pancreatic fibrosis and mediate β-cell apoptosis. In T2DM, quiescent pancreatic stellate cells may become activated, thereby contributing to pancreatic cancer development.

The positive association between T2DM and endometrial cancer risk is likely mediated by hyperinsulinemia and dysregulated endogenous hormone metabolism. Elevated levels of insulin and insulin-like growth factor 1 stimulate cell proliferation and inhibit apoptosis, effects that are particularly pronounced in metabolically active cancers such as endometrial cancer (60). Aberrant activation of insulin signaling pathways may enhance the metabolic activity and invasive capacity of endometrial cancer cells. Furthermore, hyperinsulinemia can reduce sex hormone-binding globulin levels, leading to increased circulating concentrations of free estrogen, which in turn may elevate cancer risk by promoting endometrial cell proliferation.

Inverse associations between T2DM and the risks of melanoma, gastric cancer, and esophageal cancer were observed in this study. This finding is supported by multiple lines of epidemiological evidence. An Australian nationwide study also reported a decreased risk of melanoma in T2DM patients (61). A Swedish study encompassing 125,126 individuals with T2DM also demonstrated a lower incidence of melanoma in this population compared to the general population (62). Regarding gastric cancer, Sieri et al. reported that diabetes was associated with a reduced risk, a finding consistent with the present study’s conclusions (63). However, studies from certain countries have failed to detect a significant association between T2DM and gastric cancer, and some meta-analyses have even suggested a significantly increased risk of gastric cancer in patients with T2DM, indicating that findings in this area remain controversial (64).

For cancer types that did not show significant associations in the present study, such as colorectal cancer, prostate cancer, and liver cancer, previous research has yielded somewhat inconsistent conclusions. For instance, a study focusing on African Americans indicated that individuals with T2DM had a higher risk of colorectal cancer than nondiabetic individuals (65). Other studies have suggested that T2DM might be associated with a reduced risk of prostate cancer. Davila et al. reported that patients with diabetes had a more than twofold increased risk of liver cancer compared to nondiabetic individuals (66). A meta-analysis indicated that T2DM could increase the risk of kidney cancer (67).

These discrepancies may arise from several factors. First, traditional observational studies are susceptible to confounding by lifestyle factors such as obesity, smoking, and alcohol consumption; the associations reported may not reflect true causal effects. Second, the impact of T2DM is highly heterogeneous across cancer types, with effect sizes varying considerably depending on tissue of origin and underlying carcinogenic mechanisms. For example, although hyperinsulinemia could theoretically promote lung tumorigenesis, the strong effect of smoking may mask the independent contribution of metabolic factors. In liver cancer, the influence of major etiological factors such as viral hepatitis and aflatoxin exposure likely overshadows any effect of T2DM. Additionally, competing risks offer a potential statistical explanation. Individuals with T2DM may die prematurely from other causes, such as cardiovascular events, before reaching the age at which cancer typically develops, potentially leading to spurious “protective effects” in statistical analyses.

The confounding effect of antidiabetic drugs also warrants consideration. Multiple studies have indicated that certain antidiabetic drugs possess potential anticancer properties. Metformin, a first-line therapy for T2DM, has been associated not only with a reduced incidence of several cancers, including pancreatic, endometrial, esophageal, and colon cancers, but also with improved prognosis in patients with breast cancer and prolonged survival in patients with glioblastoma (68, 69). A meta-analysis demonstrated significantly lower overall cancer incidence and cancer-related mortality among metformin users (70). Its mechanisms of action may involve modulation of the tumor immune microenvironment, activation of AMP-activated protein kinase, interference with the mTOR pathway, inhibition of tumor cell proliferation, and induction of apoptosis (71, 72).

Research suggests that glucagon-like peptide-1 receptor agonists may reduce overall cancer risk [1629], delay prostate cancer progression, inhibit pancreatic cancer cell proliferation, and lower the risk of gastrointestinal cancers (6, 8, 73). However, they may also be associated with an increased risk of thyroid cancer (74, 75). Chou et al. indicated that sodium-glucose cotransporter 2 inhibitors were associated with a reduced risk of gastric cancer (76). Canagliflozin may decrease gastrointestinal cancer risk through mechanisms such as inhibiting glucose uptake by cancer cells, inducing cell necrosis, and mitigating inflammatory responses. Although the genetic instruments used in the MR studies included in this analysis were designed to proxy T2DM, the influence of antidiabetic medications on cancer risk may not have been entirely eliminated. Beyond confounding by medication, detection bias may also influence results. Individuals with T2DM typically have more frequent medical encounters, which could lead to earlier detection and intervention of precancerous lesions, thereby reducing the risk of cancer diagnosis; however, direct evidence supporting this mechanism is lacking.

Notably, significant heterogeneity was observed for certain cancer types in this meta-analysis, such as pancreatic cancer and melanoma. This heterogeneity may stem from several sources. First, differences exist among MR studies in the selection of instrumental variables, including the number of SNPs, selection criteria, and whether pleiotropy correction was performed, potentially leading to variations in causal estimates. Second, while the included studies predominantly involved European populations, some included Asian and other populations; genetic background and T2DM phenotypic differences across ancestries may influence causal effects on cancer. Third, although we primarily extracted results based on the IVW method, the approaches used to handle potential pleiotropy, including whether methods such as MR-PRESSO were employed to exclude outliers, varied across studies. Fourth, T2DM itself is a heterogeneous condition, and its different subtypes or clinical characteristics, such as age at onset and BMI, may be associated with varying strengths of effect for specific cancers, whereas most current studies have analyzed T2DM as a uniform entity. Despite the presence of heterogeneity, leave-one-out analyses indicated that no single study exerted a decisive influence on the pooled result for pancreatic cancer, supporting the robustness of this finding to some extent. However, fully elucidating the sources of this heterogeneity at the current data level remains challenging, underscoring the need for more refined study designs in the future.

This study has several limitations. First, due to the authors’ language restrictions, only English-language publications were included, which may have introduced language bias and potentially omitted relevant studies published in other languages. Second, although we assessed publication bias using methods such as Egger’s test, its potential impact cannot be entirely excluded. Funnel plots and Egger’s tests suggested publication bias in the analyses of five cancers, which could lead to overestimation of pooled effect sizes. After applying the trim-and-fill method for correction, the pooled results did not change substantially, indicating that the overall findings were relatively robust. Third, this study focused exclusively on the association between T2DM and cancer, without distinguishing different stages of diabetes progression or including analyses of type 1 diabetes or other diabetes types in relation to cancer. Additionally, associations between biomarkers such as hemoglobin A1c, insulin, and insulin-like growth factor 1 and cancer were not evaluated. Fourth, we were unable to perform stratified analyses based on medication history, including type, dose, and duration of antidiabetic drugs, because the original studies did not provide sufficient data. In addition, we could not further explore potential heterogeneity in the T2DM-cancer association by age, such as between elderly and younger populations. Nor could we examine heterogeneity by ancestry. These limitations reduce the precision of our interpretations to some extent. The inability to account for medication use creates a challenge in interpretation. It becomes difficult to determine whether the observed associations reflect the biological effects of diabetes itself, such as hyperglycemia or insulin resistance, or instead arise from the protective or pathogenic effects of specific antidiabetic drugs, including metformin with its known anticancer properties. This limitation potentially confounds causal inference. Furthermore, differences in genetic background, lifestyle habits, and metabolic characteristics across age groups and ancestries may lead to varying susceptibility to T2DM and cancer risk; the lack of stratified analyses precluded identification of these potential effect modifiers and may have masked true strong associations or null findings in specific subgroups, limiting the overall conclusions to an “average effect” and hindering precise guidance for clinical screening and prevention strategies in diverse populations.

## 5 Conclusions

In summary, this meta-analysis demonstrates that T2DM has potential causal associations with multiple cancer types. Specifically, T2DM was associated with an increased risk of pancreatic cancer and endometrial cancer, while exhibiting inverse associations with the risks of gastric cancer, melanoma, and esophageal cancer. Furthermore, no significant causal relationships were observed between T2DM and bladder cancer, prostate cancer, colorectal cancer, liver cancer, lung cancer, kidney cancer, glioblastoma, ovarian cancer, oral cancer, or lymphoma. This study provides a genetic basis for understanding the role of T2DM in carcinogenesis and offers valuable insights for developing cancer type-specific prevention strategies.

## Data Availability

The original contributions presented in the study are included in the article/**Supplementary Material**. Further inquiries can be directed to the corresponding author.
