# Supplementary material for: Type 2 diabetes mellitus and cancer: A systematic review and meta-analysis of Mendelian randomization studies
Source: Front Endocrinol (Lausanne). 2026 Apr 15;17:1713815. doi: 10.3389/fendo.2026.1713815 (PMC13124634; doi:10.3389/fendo.2026.1713815)
Supplement: Supplementary file 1 [file DataSheet1.docx]

Supplemental Table 1. PRISMA 2020 Checklist

| **Section and Topic** | **Item #** | **Checklist item** | **Location where item is reported** |
| --- | --- | --- | --- |
| **TITLE** | | |  |
| Title | 1 | Identify the report as a systematic review. | Title |
| **ABSTRACT** | | |  |
| Abstract | 2 | See the PRISMA 2020 for Abstracts checklist. | Abstract |
| **INTRODUCTION** | | |  |
| Rationale | 3 | Describe the rationale for the review in the context of existing knowledge. | Introduction |
| Objectives | 4 | Provide an explicit statement of the objective(s) or question(s) the review addresses. | Introduction |
| **METHODS** | | |  |
| Eligibility criteria | 5 | Specify the inclusion and exclusion criteria for the review and how studies were grouped for the syntheses. | Materials and methods |
| Information sources | 6 | Specify all databases, registers, websites, organisations, reference lists and other sources searched or consulted to identify studies. Specify the date when each source was last searched or consulted. | Materials and methods |
| Search strategy | 7 | Present the full search strategies for all databases, registers and websites, including any filters and limits used. | Table S2-7 |
| Selection process | 8 | Specify the methods used to decide whether a study met the inclusion criteria of the review, including how many reviewers screened each record and each report retrieved, whether they worked independently, and if applicable, details of automation tools used in the process. | Materials and methods |
| Data collection process | 9 | Specify the methods used to collect data from reports, including how many reviewers collected data from each report, whether they worked independently, any processes for obtaining or confirming data from study investigators, and if applicable, details of automation tools used in the process. | Materials and methods |
| Data items | 10a | List and define all outcomes for which data were sought. Specify whether all results that were compatible with each outcome domain in each study were sought (e.g. for all measures, time points, analyses), and if not, the methods used to decide which results to collect. | Materials and methods |
|  | 10b | List and define all other variables for which data were sought (e.g. participant and intervention characteristics, funding sources). Describe any assumptions made about any missing or unclear information. | Materials and methods |
| Study risk of bias assessment | 11 | Specify the methods used to assess risk of bias in the included studies, including details of the tool(s) used, how many reviewers assessed each study and whether they worked independently, and if applicable, details of automation tools used in the process. | Materials and methods |
| Effect measures | 12 | Specify for each outcome the effect measure(s) (e.g. risk ratio, mean difference) used in the synthesis or presentation of results. | Materials and methods |
| Synthesis methods | 13a | Describe the processes used to decide which studies were eligible for each synthesis (e.g. tabulating the study intervention characteristics and comparing against the planned groups for each synthesis (item #5)). | Materials and methods |
|  | 13b | Describe any methods required to prepare the data for presentation or synthesis, such as handling of missing summary statistics, or data conversions. | Materials and methods |
|  | 13c | Describe any methods used to tabulate or visually display results of individual studies and syntheses. | Materials and methods |
|  | 13d | Describe any methods used to synthesize results and provide a rationale for the choice(s). If meta-analysis was performed, describe the model(s), method(s) to identify the presence and extent of statistical heterogeneity, and software package(s) used. | Materials and methods |
|  | 13e | Describe any methods used to explore possible causes of heterogeneity among study results (e.g. subgroup analysis, meta-regression). | Materials and methods |
|  | 13f | Describe any sensitivity analyses conducted to assess robustness of the synthesized results. | Materials and methods |
| Reporting bias assessment | 14 | Describe any methods used to assess risk of bias due to missing results in a synthesis (arising from reporting biases). | Materials and methods |
| Certainty assessment | 15 | Describe any methods used to assess certainty (or confidence) in the body of evidence for an outcome. | Materials and methods |


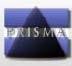
 **PRISMA 2020 Checklist**

| **Section and Topic** | **Item #** | **Checklist item** | **Location where item is reported** |
| --- | --- | --- | --- |
| **RESULTS** | |  |  |
| Study selection | 16a | Describe the results of the search and selection process, from the number of records identified in the search to the number of studies included in the review, ideally using a flow diagram. | Results, Figure 1 |
|  | 16b | Cite studies that might appear to meet the inclusion criteria, but which were excluded, and explain why they were excluded. | Results |
| Study characteristics | 17 | Cite each included study and present its characteristics. | Results, Table 1 |
| Risk of bias in studies | 18 | Present assessments of risk of bias for each included study. | Results, Table 2-3 |
| Results of individual studies | 19 | For all outcomes, present, for each study: (a) summary statistics for each group (where appropriate) and (b) an effect estimate and its precision (e.g. confidence/credible interval), ideally using structured tables or plots. | Results |
| Results of syntheses | 20a | For each synthesis, briefly summarise the characteristics and risk of bias among contributing studies. | Results |
|  | 20b | Present results of all statistical syntheses conducted. If meta-analysis was done, present for each the summary estimate and its precision (e.g.  confidence/credible interval) and measures of statistical heterogeneity. If comparing groups, describe the direction of the effect. | Results |
|  | 20c | Present results of all investigations of possible causes of heterogeneity among study results. | Results |
|  | 20d | Present results of all sensitivity analyses conducted to assess the robustness of the synthesized results. | Results |
| Reporting biases | 21 | Present assessments of risk of bias due to missing results (arising from reporting biases) for each synthesis assessed. | Results |
| Certainty of evidence | 22 | Present assessments of certainty (or confidence) in the body of evidence for each outcome assessed. | NA |
| **DISCUSSION** | |  |  |
| Discussion | 23a | Provide a general interpretation of the results in the context of other evidence. | Discussion |
|  | 23b | Discuss any limitations of the evidence included in the review. | Discussion |
|  | 23c | Discuss any limitations of the review processes used. | Discussion |
|  | 23d | Discuss implications of the results for practice, policy, and future research. | Discussion |
| **OTHER INFORMATION** | |  |  |
| Registration and protocol | 24a | Provide registration information for the review, including register name and registration number, or state that the review was not registered. | Materials and methods |
|  | 24b | Indicate where the review protocol can be accessed, or state that a protocol was not prepared. | Materials and methods |
|  | 24c | Describe and explain any amendments to information provided at registration or in the protocol. | NA |
| Support | 25 | Describe sources of financial or non-financial support for the review, and the role of the funders or sponsors in the review. | Funding |
| Competing  interests | 26 | Declare any competing interests of review authors. | Conflict of interest |
| Availability of data, code and other materials | 27 | Report which of the following are publicly available and where they can be found: template data collection forms; data extracted from included studies; data used for all analyses; analytic code; any other materials used in the review. | Data ability statement，Code S1 |

*From:*  Page MJ, McKenzie JE, Bossuyt PM, Boutron I, Hoffmann TC, Mulrow CD, et al. The PRISMA 2020 statement: an updated guideline for reporting systematic reviews. BMJ 2021;372:n71. doi:

10.1136/bmj.n71

**Supplementary Table 2. Search strategy for the Scopus database**

| 1 | (“Diabetes Mellitus, Type 2”) OR (“Diabetes Mellitus, Stable”) OR (“Stable Diabetes Mellitus”) OR (“Diabetes Mellitus, Noninsulin Dependent”) OR (“Diabetes Mellitus, Adult-Onset”) OR (“Adult-Onset Diabetes Mellitus”) OR (“Diabetes Mellitus, Adult Onset”) OR (“Diabetes Mellitus, Ketosis-Resistant”) OR (“Diabetes Mellitus, Ketosis Resistant”) OR (“Ketosis-Resistant Diabetes Mellitus”) OR (“Diabetes Mellitus, Non Insulin Dependent”) OR (“Diabetes Mellitus, Non-Insulin-Dependent”) OR (“Non-Insulin-Dependent Diabetes Mellitus”) OR (“Diabetes Mellitus, Type II”) OR (“NIDDM”) OR (“Diabetes Mellitus, Maturity-Onset”) OR (“Diabetes Mellitus, Maturity Onset”) OR (“Maturity-Onset Diabetes Mellitus”) OR (“Maturity Onset Diabetes Mellitus”) OR (“MODY”) OR (“Diabetes Mellitus, Slow-Onset”) OR (“Diabetes Mellitus, Slow Onset”) OR (“Slow-Onset Diabetes Mellitus”) OR (“Type 2 Diabetes Mellitus”) OR (“Noninsulin-Dependent Diabetes Mellitus”) OR (“Noninsulin Dependent Diabetes Mellitus”) OR (“Maturity-Onset Diabetes”) OR (“Diabetes, Maturity-Onset”) OR (“Maturity Onset Diabetes”) OR (“Type 2 Diabetes”) OR (“Diabetes, Type 2”) OR (“Diabetes Mellitus, Noninsulin-Dependent”) | 384,321 |
| --- | --- | --- |
| 2 | (“Neoplasms”) OR (“Tumors”) OR (“Neoplasia”) OR (“Neoplasias”) OR (“Neoplasm”) OR (“Tumor”) OR (“Cancer”) OR (“Cancers”) OR (“Malignant Neoplasm”) OR (“Malignancy”) OR (“Malignancies”) OR (“Malignant Neoplasms”) OR (“Neoplasm, Malignant”) OR (“Neoplasms, Malignant”) OR (“Benign Neoplasms”) OR (“Neoplasms, Benign”) OR (“Neoplasm, Benign”) OR (“Benign Neoplasm”) | 6,607,202 |
| 3 | (“Mendelian Randomization”) OR (“Analysis, Mendelian Randomization”) OR (“Mendelian randomisation”) OR (“Mendelian Randomisation”) OR (“genetic instrumental”) OR (“genetic instrument”) OR (“instrumental variable”) | 37,262 |
| 4 | #1 AND #2 AND #3 | 330 |

**Supplementary Table 3. Search strategy for PubMed**

| 1 | “Diabetes Mellitus, Type 2”[Mesh] | 190,360 |
| --- | --- | --- |
| 2 | ((((((((((((((((((((((((((((((Diabetes Mellitus, Stable [Title/Abstract]) OR (Stable Diabetes Mellitus [Title/Abstract])) OR (Diabetes Mellitus, Noninsulin Dependent [Title/Abstract])) OR (Diabetes Mellitus, Adult-Onset [Title/Abstract])) OR (Adult-Onset Diabetes Mellitus [Title/Abstract])) OR (Diabetes Mellitus, Adult Onset [Title/Abstract])) OR (Diabetes Mellitus, Ketosis-Resistant [Title/Abstract])) OR (Diabetes Mellitus, Ketosis Resistant [Title/Abstract])) OR (Ketosis-Resistant Diabetes Mellitus [Title/Abstract])) OR (Diabetes Mellitus, Non Insulin Dependent [Title/Abstract])) OR (Diabetes Mellitus, Non-Insulin-Dependent [Title/Abstract])) OR (Non-Insulin-Dependent Diabetes Mellitus [Title/Abstract])) OR (Diabetes Mellitus, Type II [Title/Abstract])) OR (NIDDM [Title/Abstract])) OR (Diabetes Mellitus, Maturity-Onset [Title/Abstract])) OR (Diabetes Mellitus, Maturity Onset [Title/Abstract])) OR (Maturity-Onset Diabetes Mellitus [Title/Abstract])) OR (Maturity Onset Diabetes Mellitus [Title/Abstract])) OR (MODY [Title/Abstract])) OR (Diabetes Mellitus, Slow-Onset [Title/Abstract])) OR (Diabetes Mellitus, Slow Onset [Title/Abstract])) OR (Slow-Onset Diabetes Mellitus [Title/Abstract])) OR (Type 2 Diabetes Mellitus [Title/Abstract])) OR (Noninsulin-Dependent Diabetes Mellitus [Title/Abstract])) OR (Noninsulin Dependent Diabetes Mellitus [Title/Abstract])) OR (Maturity-Onset Diabetes [Title/Abstract])) OR (Diabetes, Maturity-Onset [Title/Abstract])) OR (Maturity Onset Diabetes [Title/Abstract])) OR (Type 2 Diabetes [Title/Abstract])) OR (Diabetes, Type 2 [Title/Abstract])) OR (Diabetes Mellitus, Noninsulin-Dependent [Title/Abstract]) | 208,577 |
| 3 | (“Diabetes Mellitus, Type 2” [Mesh]) OR (((((((((((((((((((((((((((((((Diabetes Mellitus, Stable [Title/Abstract]) OR (Stable Diabetes Mellitus [Title/Abstract])) OR (Diabetes Mellitus, Noninsulin Dependent [Title/Abstract])) OR (Diabetes Mellitus, Adult-Onset [Title/Abstract])) OR (Adult-Onset Diabetes Mellitus [Title/Abstract])) OR (Diabetes Mellitus, Adult Onset [Title/Abstract])) OR (Diabetes Mellitus, Ketosis-Resistant [Title/Abstract])) OR (Diabetes Mellitus, Ketosis Resistant [Title/Abstract])) OR (Ketosis-Resistant Diabetes Mellitus [Title/Abstract])) OR (Diabetes Mellitus, Non Insulin Dependent [Title/Abstract])) OR (Diabetes Mellitus, Non-Insulin-Dependent [Title/Abstract])) OR (Non-Insulin-Dependent Diabetes Mellitus [Title/Abstract])) OR (Diabetes Mellitus, Type II [Title/Abstract])) OR (NIDDM [Title/Abstract])) OR (Diabetes Mellitus, Maturity-Onset [Title/Abstract])) OR (Diabetes Mellitus, Maturity Onset [Title/Abstract])) OR (Maturity-Onset Diabetes Mellitus [Title/Abstract])) OR (Maturity Onset Diabetes Mellitus [Title/Abstract])) OR (MODY [Title/Abstract])) OR (Diabetes Mellitus, Slow-Onset [Title/Abstract])) OR (Diabetes Mellitus, Slow Onset [Title/Abstract])) OR (Slow-Onset Diabetes Mellitus [Title/Abstract])) OR (Type 2 Diabetes Mellitus [Title/Abstract])) OR (Noninsulin-Dependent Diabetes Mellitus [Title/Abstract])) OR (Noninsulin Dependent Diabetes Mellitus [Title/Abstract])) OR (Maturity-Onset Diabetes [Title/Abstract])) OR (Diabetes, Maturity-Onset [Title/Abstract])) OR (Maturity Onset Diabetes [Title/Abstract])) OR (Type 2 Diabetes [Title/Abstract])) OR (Diabetes, Type 2 [Title/Abstract])) OR (Diabetes Mellitus, Noninsulin-Dependent [Title/Abstract])) | 263,470 |
| 4 | “Neoplasms”[Mesh] | 4,117,881 |
| 5 | ((((((((((((((((Tumors [Title/Abstract]) OR (Neoplasia [Title/Abstract])) OR (Neoplasias [Title/Abstract])) OR (Neoplasm [Title/Abstract])) OR (Tumor [Title/Abstract])) OR (Cancer [Title/Abstract])) OR (Cancers [Title/Abstract])) OR (Malignant Neoplasm [Title/Abstract])) OR (Malignancy [Title/Abstract])) OR (Malignancies [Title/Abstract])) OR (Malignant Neoplasms [Title/Abstract])) OR (Neoplasm, Malignant [Title/Abstract])) OR (Neoplasms, Malignant [Title/Abstract])) OR (Benign Neoplasms [Title/Abstract])) OR (Neoplasms, Benign [Title/Abstract])) OR (Neoplasm, Benign [Title/Abstract])) OR (Benign Neoplasm [Title/Abstract]) | 3,802,802 |
| 6 | (“Neoplasms” [Mesh]) OR (((((((((((((((((Tumors [Title/Abstract]) OR (Neoplasia [Title/Abstract])) OR (Neoplasias [Title/Abstract])) OR (Neoplasm [Title/Abstract])) OR (Tumor [Title/Abstract])) OR (Cancer [Title/Abstract])) OR (Cancers [Title/Abstract])) OR (Malignant Neoplasm [Title/Abstract])) OR (Malignancy [Title/Abstract])) OR (Malignancies [Title/Abstract])) OR (Malignant Neoplasms [Title/Abstract])) OR (Neoplasm, Malignant [Title/Abstract])) OR (Neoplasms, Malignant [Title/Abstract])) OR (Benign Neoplasms [Title/Abstract])) OR (Neoplasms, Benign [Title/Abstract])) OR (Neoplasm, Benign [Title/Abstract])) OR (Benign Neoplasm [Title/Abstract])) | 5,334,193 |
| 7 | “Mendelian Randomization Analysis”[Mesh] | 10,134 |
| 8 | (((((Analysis, Mendelian Randomization [Title/Abstract]) OR (Mendelian randomisation [Title/Abstract])) OR (Mendelian Randomisation [Title/Abstract])) OR (genetic instrumental [Title/Abstract])) OR (genetic instrument [Title/Abstract])) OR (instrumental variable [Title/Abstract]) | 4,718 |
| 9 | (“Mendelian Randomization Analysis” [Mesh]) OR ((((((Analysis, Mendelian Randomization [Title/Abstract]) OR (Mendelian randomisation [Title/Abstract])) OR (Mendelian Randomisation [Title/Abstract])) OR (genetic instrumental [Title/Abstract])) OR (genetic instrument [Title/Abstract])) OR (instrumental variable [Title/Abstract])) | 13,532 |
| 10 | (((“Diabetes Mellitus, Type 2” [Mesh]) OR (((((((((((((((((((((((((((((((Diabetes Mellitus, Stable [Title/Abstract]) OR (Stable Diabetes Mellitus [Title/Abstract])) OR (Diabetes Mellitus, Noninsulin Dependent [Title/Abstract])) OR (Diabetes Mellitus, Adult-Onset [Title/Abstract])) OR (Adult-Onset Diabetes Mellitus [Title/Abstract])) OR (Diabetes Mellitus, Adult Onset [Title/Abstract])) OR (Diabetes Mellitus, Ketosis-Resistant [Title/Abstract])) OR (Diabetes Mellitus, Ketosis Resistant [Title/Abstract])) OR (Ketosis-Resistant Diabetes Mellitus [Title/Abstract])) OR (Diabetes Mellitus, Non Insulin Dependent [Title/Abstract])) OR (Diabetes Mellitus, Non-Insulin-Dependent [Title/Abstract])) OR (Non-Insulin-Dependent Diabetes Mellitus [Title/Abstract])) OR (Diabetes Mellitus, Type II [Title/Abstract])) OR (NIDDM [Title/Abstract])) OR (Diabetes Mellitus, Maturity-Onset [Title/Abstract])) OR (Diabetes Mellitus, Maturity Onset [Title/Abstract])) OR (Maturity-Onset Diabetes Mellitus [Title/Abstract])) OR (Maturity Onset Diabetes Mellitus [Title/Abstract])) OR (MODY [Title/Abstract])) OR (Diabetes Mellitus, Slow-Onset [Title/Abstract])) OR (Diabetes Mellitus, Slow Onset [Title/Abstract])) OR (Slow-Onset Diabetes Mellitus [Title/Abstract])) OR (Type 2 Diabetes Mellitus [Title/Abstract])) OR (Noninsulin-Dependent Diabetes Mellitus [Title/Abstract])) OR (Noninsulin Dependent Diabetes Mellitus [Title/Abstract])) OR (Maturity-Onset Diabetes [Title/Abstract])) OR (Diabetes, Maturity-Onset [Title/Abstract])) OR (Maturity Onset Diabetes [Title/Abstract])) OR (Type 2 Diabetes [Title/Abstract])) OR (Diabetes, Type 2 [Title/Abstract])) OR (Diabetes Mellitus, Noninsulin-Dependent [Title/Abstract]))) AND ((“Neoplasms” [Mesh]) OR (((((((((((((((((Tumors [Title/Abstract]) OR (Neoplasia [Title/Abstract])) OR (Neoplasias [Title/Abstract])) OR (Neoplasm [Title/Abstract])) OR (Tumor [Title/Abstract])) OR (Cancer [Title/Abstract])) OR (Cancers [Title/Abstract])) OR (Malignant Neoplasm [Title/Abstract])) OR (Malignancy [Title/Abstract])) OR (Malignancies [Title/Abstract])) OR (Malignant Neoplasms [Title/Abstract])) OR (Neoplasm, Malignant [Title/Abstract])) OR (Neoplasms, Malignant [Title/Abstract])) OR (Benign Neoplasms [Title/Abstract])) OR (Neoplasms, Benign [Title/Abstract])) OR (Neoplasm, Benign [Title/Abstract])) OR (Benign Neoplasm [Title/Abstract])))) AND ((“Mendelian Randomization Analysis” [Mesh]) OR ((((((Analysis, Mendelian Randomization [Title/Abstract]) OR (Mendelian randomisation [Title/Abstract])) OR (Mendelian Randomisation [Title/Abstract])) OR (genetic instrumental [Title/Abstract])) OR (genetic instrument [Title/Abstract])) OR (instrumental variable [Title/Abstract]))) | 133 |

**Supplementary Table 4. Search strategy for the Cochrane Library**

| 1 | Diabetes Mellitus, Type 2 | 75,782 |
| --- | --- | --- |
| 2 | (Diabetes Mellitus, Stable):ab,ti,kw OR (Stable Diabetes Mellitus):ab,ti,kw OR (Diabetes Mellitus, Noninsulin Dependent):ab,ti,kw OR (Diabetes Mellitus, Adult-Onset):ab,ti,kw OR (Adult-Onset Diabetes Mellitus):ab,ti,kw OR (Diabetes Mellitus, Adult Onset):ab,ti,kw OR (Diabetes Mellitus, Ketosis-Resistant):ab,ti,kw OR (Diabetes Mellitus, Ketosis Resistant):ab,ti,kw OR (Ketosis-Resistant Diabetes Mellitus):ab,ti,kw OR (Diabetes Mellitus, Non Insulin Dependent):ab,ti,kw OR (Diabetes Mellitus, Non-Insulin-Dependent):ab,ti,kw OR (Non-Insulin-Dependent Diabetes Mellitus):ab,ti,kw OR (Diabetes Mellitus, Type II):ab,ti,kw OR (NIDDM):ab,ti,kw OR (Diabetes Mellitus, Maturity-Onset):ab,ti,kw OR (Diabetes Mellitus, Maturity Onset):ab,ti,kw OR (Maturity-Onset Diabetes Mellitus):ab,ti,kw OR (Maturity Onset Diabetes Mellitus):ab,ti,kw OR (MODY):ab,ti,kw OR (Diabetes Mellitus, Slow-Onset):ab,ti,kw OR (Diabetes Mellitus, Slow Onset):ab,ti,kw OR (Slow-Onset Diabetes Mellitus):ab,ti,kw OR (Type 2 Diabetes Mellitus):ab,ti,kw OR (Noninsulin-Dependent Diabetes Mellitus):ab,ti,kw OR (Noninsulin Dependent Diabetes Mellitus):ab,ti,kw OR (Maturity-Onset Diabetes):ab,ti,kw OR (Diabetes, Maturity-Onset):ab,ti,kw OR (Maturity Onset Diabetes):ab,ti,kw OR (Type 2 Diabetes):ab,ti,kw OR (Diabetes, Type 2):ab,ti,kw OR (Diabetes Mellitus, Noninsulin-Dependent):ab,ti,kw | 69,371 |
| 3 | #1 OR #2 | 86,077 |
| 4 | Neoplasms | 110,226 |
| 5 | (Tumors):ab,ti,kw OR (Neoplasia):ab,ti,kw OR (Neoplasias):ab,ti,kw OR (Neoplasm):ab,ti,kw OR (Tumor):ab,ti,kw OR (Cancer):ab,ti,kw OR (Cancers):ab,ti,kw OR (Malignant Neoplasm):ab,ti,kw OR (Malignancy):ab,ti,kw OR (Malignancies):ab,ti,kw OR (Malignant Neoplasms):ab,ti,kw OR (Neoplasm, Malignant):ab,ti,kw OR (Neoplasms, Malignant):ab,ti,kw OR (Benign Neoplasms):ab,ti,kw OR (Neoplasms, Benign):ab,ti,kw OR (Neoplasm, Benign):ab,ti,kw OR (Benign Neoplasm):ab,ti,kw | 269,045 |
| 6 | #4 OR #5 | 280,567 |
| 7 | Mendelian Randomization | 1,754 |
| 8 | (Analysis, Mendelian Randomization):ab,ti,kw OR (Mendelian randomisation):ab,ti,kw OR (Mendelian Randomisation):ab,ti,kw OR (genetic instrumental):ab,ti,kw OR (genetic instrument):ab,ti,kw OR (instrumental variable):ab,ti,kw | 2,325 |
| 9 | #7 OR #8 | 2,340 |
| 10 | #3 AND #6 AND #9 | 31 |

**Supplementary Table 5. Search strategy for Web of Science**

| 1 | TS=(Diabetes Mellitus, Type 2 OR Diabetes Mellitus, Stable OR Stable Diabetes Mellitus OR Diabetes Mellitus, Noninsulin Dependent OR Diabetes Mellitus, Adult-Onset OR Adult-Onset Diabetes Mellitus OR Diabetes Mellitus, Adult Onset OR Diabetes Mellitus, Ketosis-Resistant OR Diabetes Mellitus, Ketosis Resistant OR Ketosis-Resistant Diabetes Mellitus OR Diabetes Mellitus, Non Insulin Dependent OR Diabetes Mellitus, Non-Insulin-Dependent OR Non-Insulin-Dependent Diabetes Mellitus OR Diabetes Mellitus, Type II OR NIDDM OR Diabetes Mellitus, Maturity-Onset OR Diabetes Mellitus, Maturity Onset OR Maturity-Onset Diabetes Mellitus OR Maturity Onset Diabetes Mellitus OR MODY OR Diabetes Mellitus, Slow-Onset OR Diabetes Mellitus, Slow Onset OR Slow-Onset Diabetes Mellitus OR Type 2 Diabetes Mellitus OR Noninsulin-Dependent Diabetes Mellitus OR Noninsulin Dependent Diabetes Mellitus OR Maturity-Onset Diabetes OR Diabetes, Maturity-Onset OR Maturity Onset Diabetes OR Type 2 Diabetes OR Diabetes, Type 2 OR Diabetes Mellitus, Noninsulin-Dependent) | 320,192 |
| --- | --- | --- |
| 2 | TS=(Neoplasms OR Tumors OR Neoplasia OR Neoplasias OR Neoplasm OR Tumor OR Cancer OR Cancers OR Malignant Neoplasm OR Malignancy OR Malignancies OR Malignant Neoplasms OR Neoplasm, Malignant OR Neoplasms, Malignant OR Benign Neoplasms OR Neoplasms, Benign OR Neoplasm, Benign OR Benign Neoplasm) | 4,938,895 |
| 3 | TS=(Mendelian Randomization OR Analysis, Mendelian Randomization OR Mendelian randomisation OR Mendelian Randomisation OR genetic instrumental OR genetic instrument OR instrumental variable) | 50,747 |
| 4 | #3 AND #2 AND #1 | 235 |

**Supplementary Table 6. Search strategy for Embase**

| 1 | (‘diabetes’/exp OR diabetes) AND mellitus, AND type AND (‘2’/exp OR 2) | 495,726 |
| --- | --- | --- |
| 2 | ‘diabetes mellitus, stable’:ab,ti OR ‘stable diabetes mellitus’:ab,ti OR ‘diabetes mellitus, noninsulin dependent’:ab,ti OR ‘diabetes mellitus, adult-onset’:ab,ti OR ‘adult-onset diabetes mellitus’:ab,ti OR ‘diabetes mellitus, adult onset’:ab,ti OR ‘diabetes mellitus, ketosis-resistant’:ab,ti OR ‘diabetes mellitus, ketosis resistant’:ab,ti OR ‘ketosis-resistant diabetes mellitus’:ab,ti OR ‘diabetes mellitus, non insulin dependent’:ab,ti OR ‘diabetes mellitus, non-insulin-dependent’:ab,ti OR ‘non-insulin-dependent diabetes mellitus’:ab,ti OR ‘diabetes mellitus, type ii’:ab,ti OR ‘niddm’:ab,ti OR ‘diabetes mellitus, maturity-onset’:ab,ti OR ‘diabetes mellitus, maturity onset’:ab,ti OR ‘maturity-onset diabetes mellitus’:ab,ti OR ‘maturity onset diabetes mellitus’:ab,ti OR ‘mody’:ab,ti OR ‘diabetes mellitus, slow-onset’:ab,ti OR ‘diabetes mellitus, slow onset’:ab,ti OR ‘slow-onset diabetes mellitus’:ab,ti OR ‘type 2 diabetes mellitus’:ab,ti OR ‘noninsulin-dependent diabetes mellitus’:ab,ti OR ‘noninsulin dependent diabetes mellitus’:ab,ti OR ‘maturity-onset diabetes’:ab,ti OR ‘diabetes, maturity-onset’:ab,ti OR ‘maturity onset diabetes’:ab,ti OR ‘type 2 diabetes’:ab,ti OR ‘diabetes, type 2’:ab,ti OR ‘diabetes mellitus, noninsulin-dependent’:ab,ti | 317,822 |
| 3 | #1 OR #2 | 527,911 |
| 4 | neoplasms | 329,031 |
| 5 | ‘tumors’:ab,ti OR ‘neoplasia’:ab,ti OR ‘neoplasias’:ab,ti OR ‘neoplasm’:ab,ti OR ‘tumor’:ab,ti OR ‘cancer’:ab,ti OR ‘cancers’:ab,ti OR ‘malignant neoplasm’:ab,ti OR ‘malignancy’:ab,ti OR ‘malignancies’:ab,ti OR ‘malignant neoplasms’:ab,ti OR ‘neoplasm, malignant’:ab,ti OR ‘neoplasms, malignant’:ab,ti OR ‘benign neoplasms’:ab,ti OR ‘neoplasms, benign’:ab,ti OR ‘neoplasm, benign’:ab,ti OR ‘benign neoplasm’:ab,ti | 5,332,692 |
| 6 | #4 OR #5 | 5,433,893 |
| 7 | mendelian AND randomization | 20,967 |
| 8 | ‘analysis, mendelian randomization’:ab,ti OR ‘mendelian randomisation’:ab,ti OR ‘genetic instrumental’:ab,ti OR ‘genetic instrument’:ab,ti OR ‘instrumental variable’:ab,ti | 5,659 |
| 9 | #7 OR #8 | 23,973 |
| 10 | #3 AND #6 AND #9 | 242 |

**Supplementary Table 7. Search strategy for Ovid MEDLINE**

| 1 | Diabetes Mellitus, Type 2 | 190,546 |
| --- | --- | --- |
| 2 | (Diabetes Mellitus, Stable OR Stable Diabetes Mellitus OR Diabetes Mellitus, Noninsulin Dependent OR Diabetes Mellitus, Adult-Onset OR Adult-Onset Diabetes Mellitus OR Diabetes Mellitus, Adult Onset OR Diabetes Mellitus, Ketosis-Resistant OR Diabetes Mellitus, Ketosis Resistant OR Ketosis-Resistant Diabetes Mellitus OR Diabetes Mellitus, Non Insulin Dependent OR Diabetes Mellitus, Non-Insulin-Dependent OR Non-Insulin-Dependent Diabetes Mellitus OR Diabetes Mellitus, Type II OR NIDDM OR Diabetes Mellitus, Maturity-Onset OR Diabetes Mellitus, Maturity Onset OR Maturity-Onset Diabetes Mellitus OR Maturity Onset Diabetes Mellitus OR MODY OR Diabetes Mellitus, Slow-Onset OR Diabetes Mellitus, Slow Onset OR Slow-Onset Diabetes Mellitus OR Type 2 Diabetes Mellitus OR Noninsulin-Dependent Diabetes Mellitus OR Noninsulin Dependent Diabetes Mellitus OR Maturity-Onset Diabetes OR Diabetes, Maturity-Onset OR Maturity Onset Diabetes OR Type 2 Diabetes OR Diabetes, Type 2 OR Diabetes Mellitus, Noninsulin-Dependent).ab,ti,kw. | 208,381 |
| 3 | #1 OR #2 | 263,252 |
| 4 | Neoplasms | 544,815 |
| 5 | (Tumors OR Neoplasia OR Neoplasias OR Neoplasm OR Tumor OR Cancer OR Cancers OR Malignant Neoplasm OR Malignancy OR Malignancies OR Malignant Neoplasms OR Neoplasm, Malignant OR Neoplasms, Malignant OR Benign Neoplasms OR Neoplasms, Benign OR Neoplasm, Benign OR Benign Neoplasm).ab,ti,kw. | 3,748,506 |
| 6 | #4 OR #5 | 3,874,835 |
| 7 | (Mendelian Randomization OR Analysis, Mendelian Randomization OR Mendelian randomisation OR Mendelian Randomisation OR genetic instrumental OR genetic instrument OR instrumental variable).ab,ti,kw. | 19,509 |
| 8 | #3 AND #6 AND #7 | 172 |

**
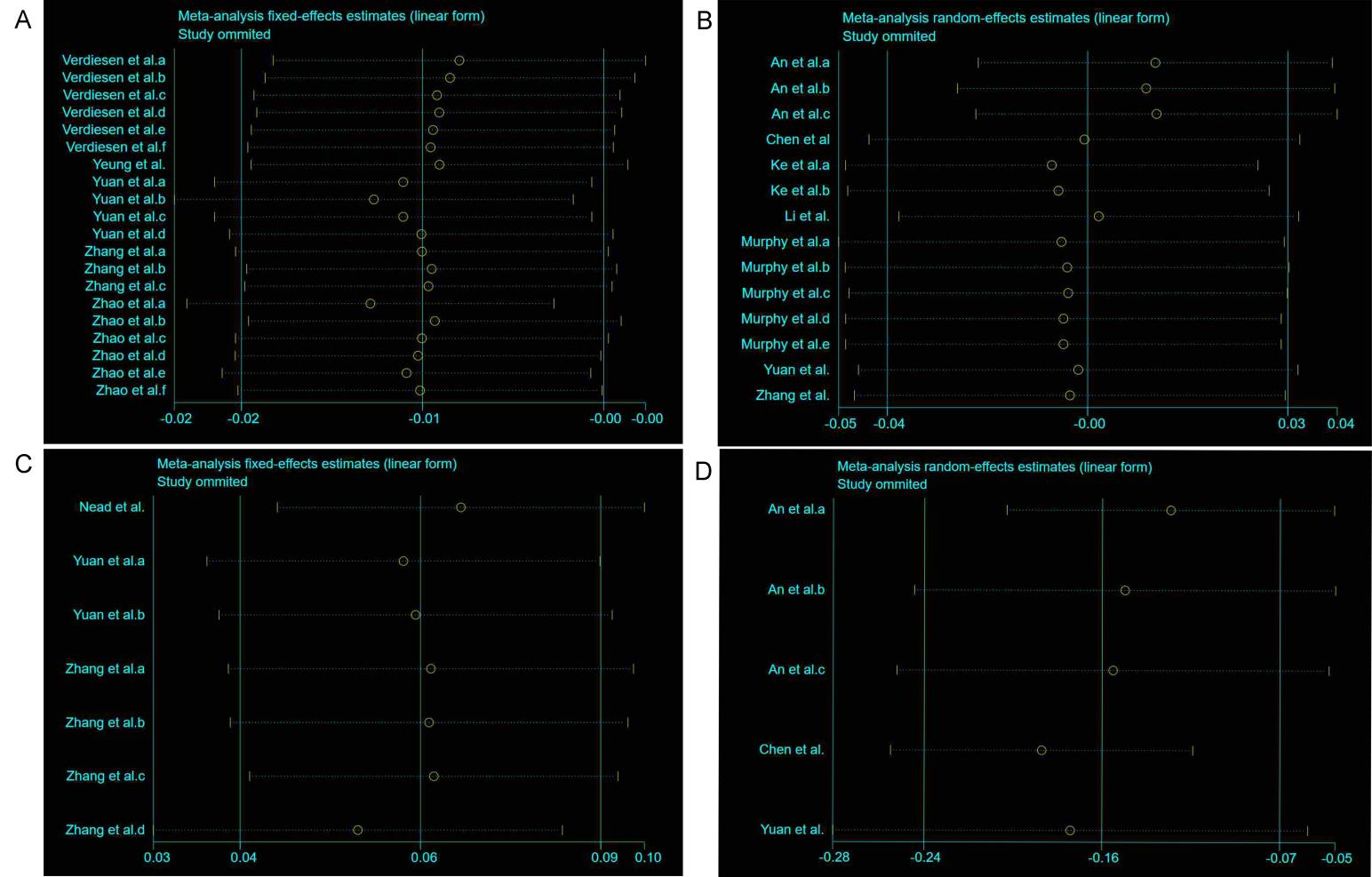
**

**Figure S1** Leave-one-out analysis for the association between type 2 diabetes mellitus and cancer risk: (A) breast cancer, (B) colorectal cancer, (C) endometrial cancer, (D) esophageal cancer.

**
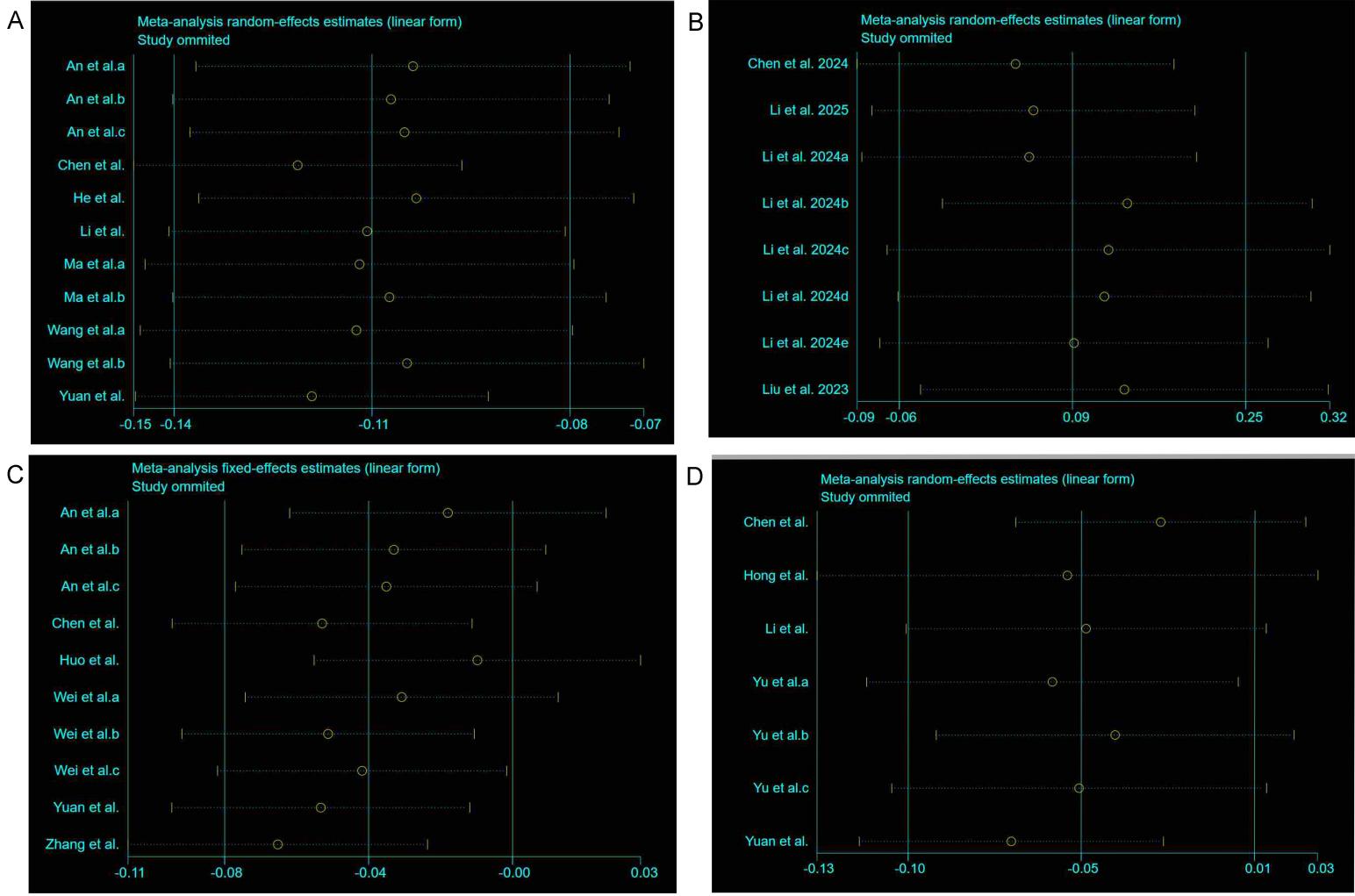
**

**Figure S2** Leave-one-out analysis for the association between type 2 diabetes mellitus and cancer risk: (A) gastric cancer, (B) glioblastoma, (C) liver cancer, (D) lung cancer.

.

**
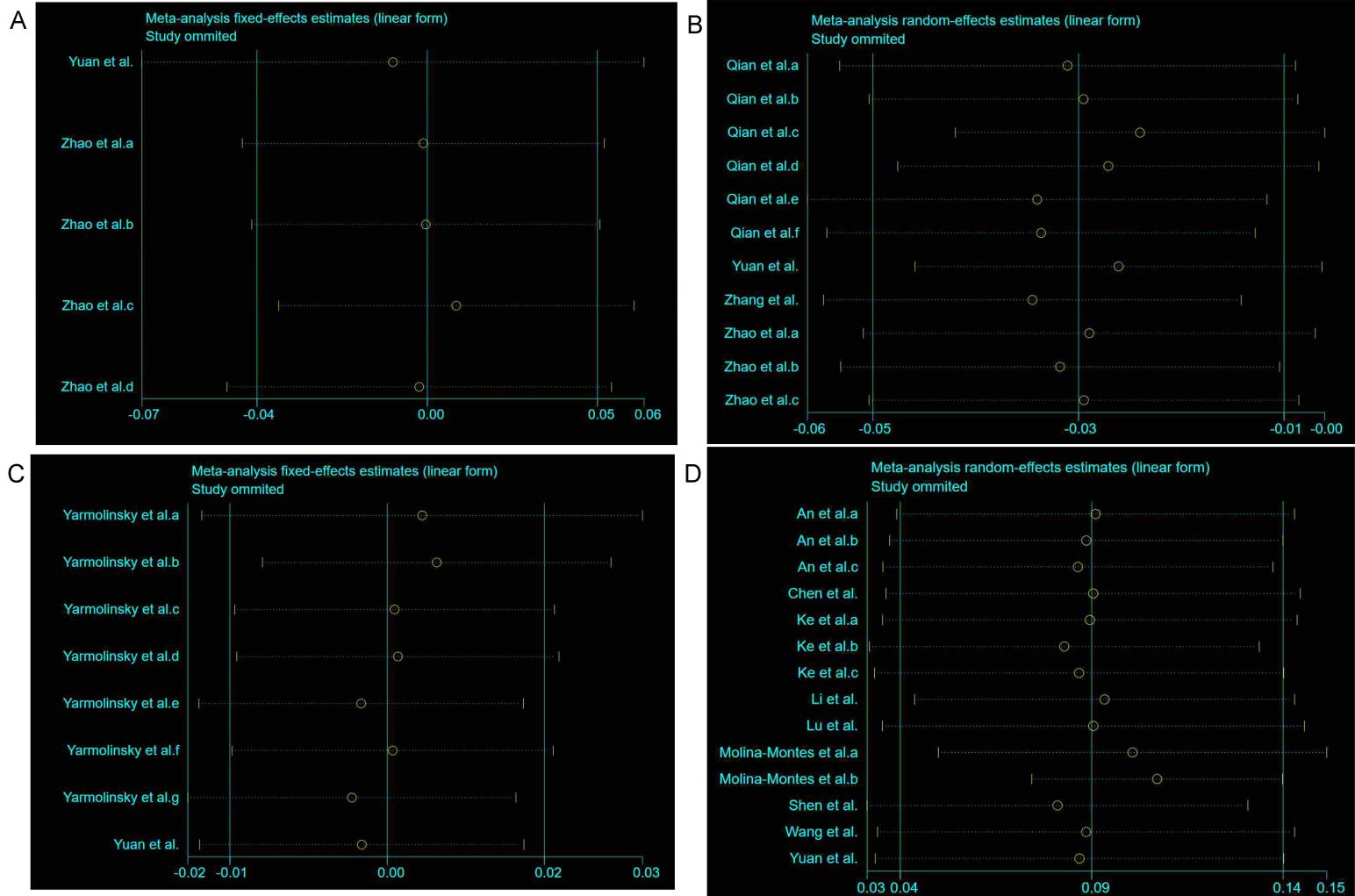
**

**Figure S3** Leave-one-out analysis for the association between type 2 diabetes mellitus and cancer risk: (A) lymphoma, (B) melanoma, (C) ovary cancer, (D) pancreatic cancer.

**
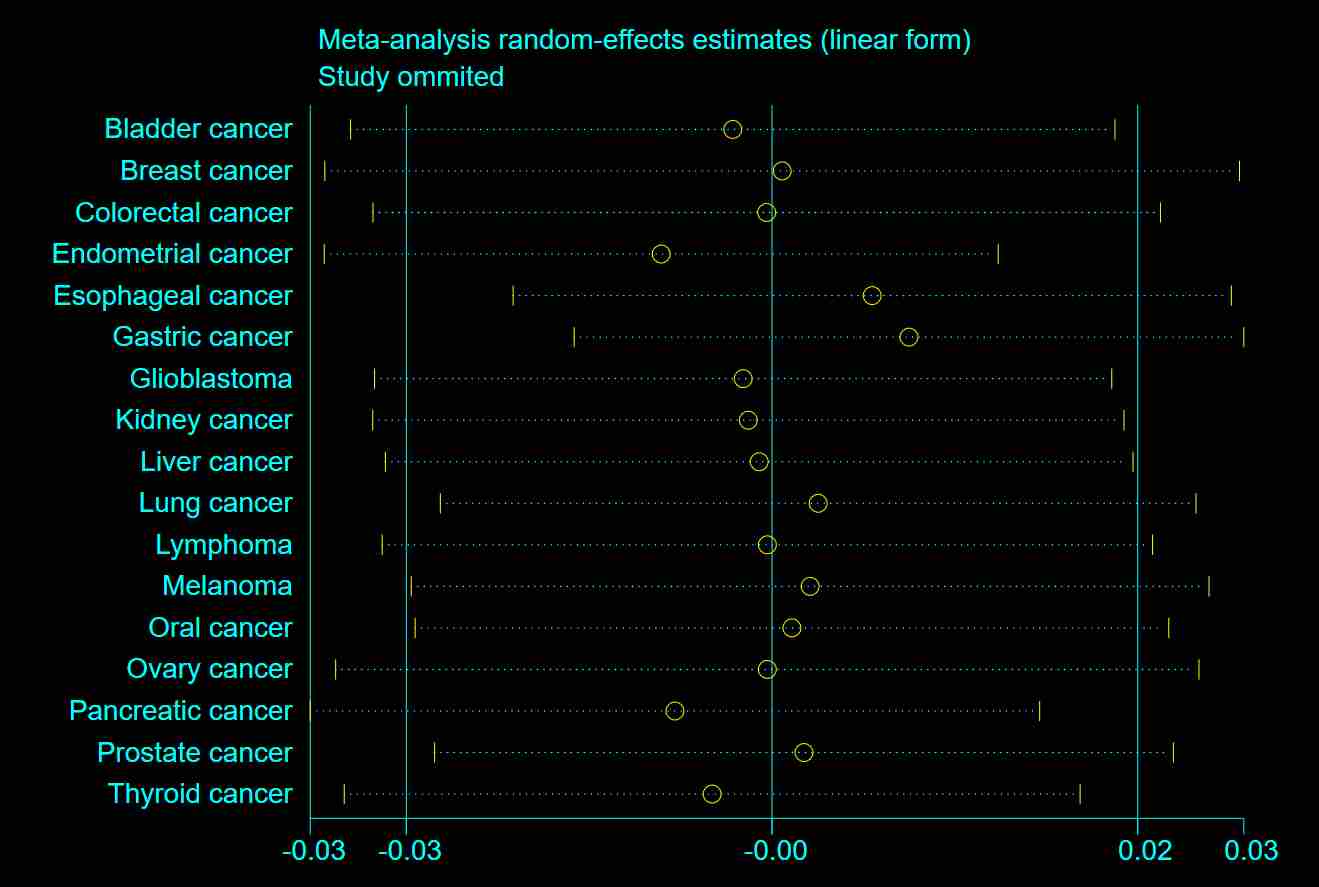
**

**Figure S4** Leave-one-out analysis for the association between type 2 diabetes mellitus and cancer risk by cancer type.

**
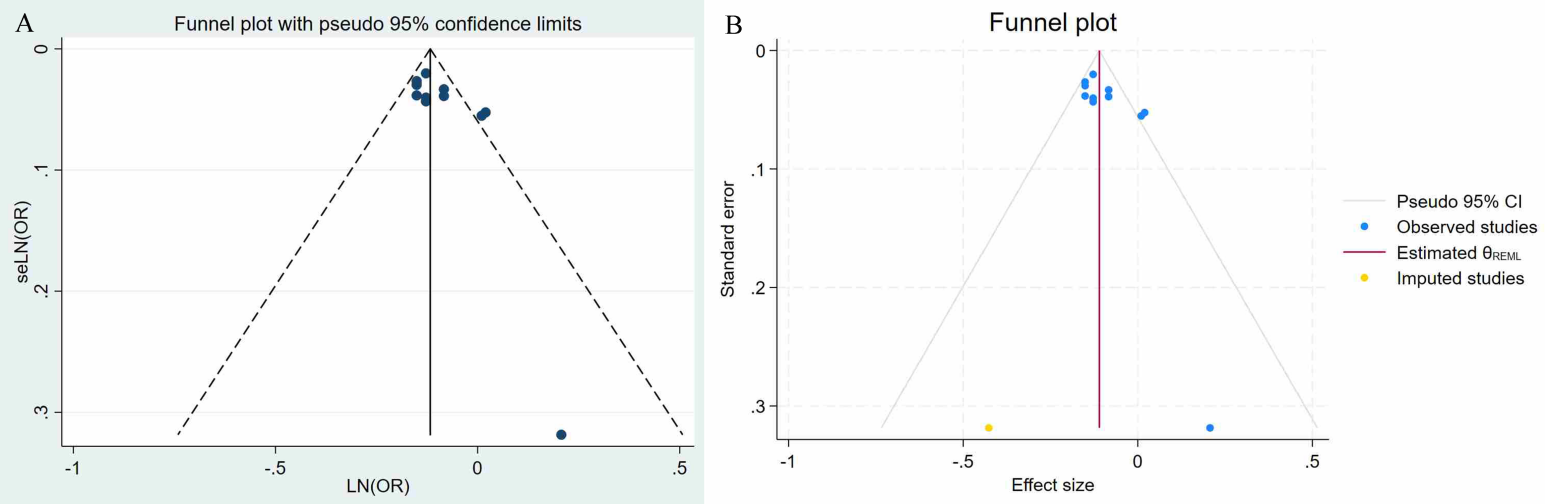
**

**Figure_S5** (A) Funnel plot suggesting potential publication bias in studies on type 2 diabetes mellitus and gastric cancer. (B) Funnel plot showing the imputed study following trim-and-fill analysis.

Abbreviations: SE,standard error; LN(OR), logarithm of the odds ratio; CI, confidence interval.

i

**
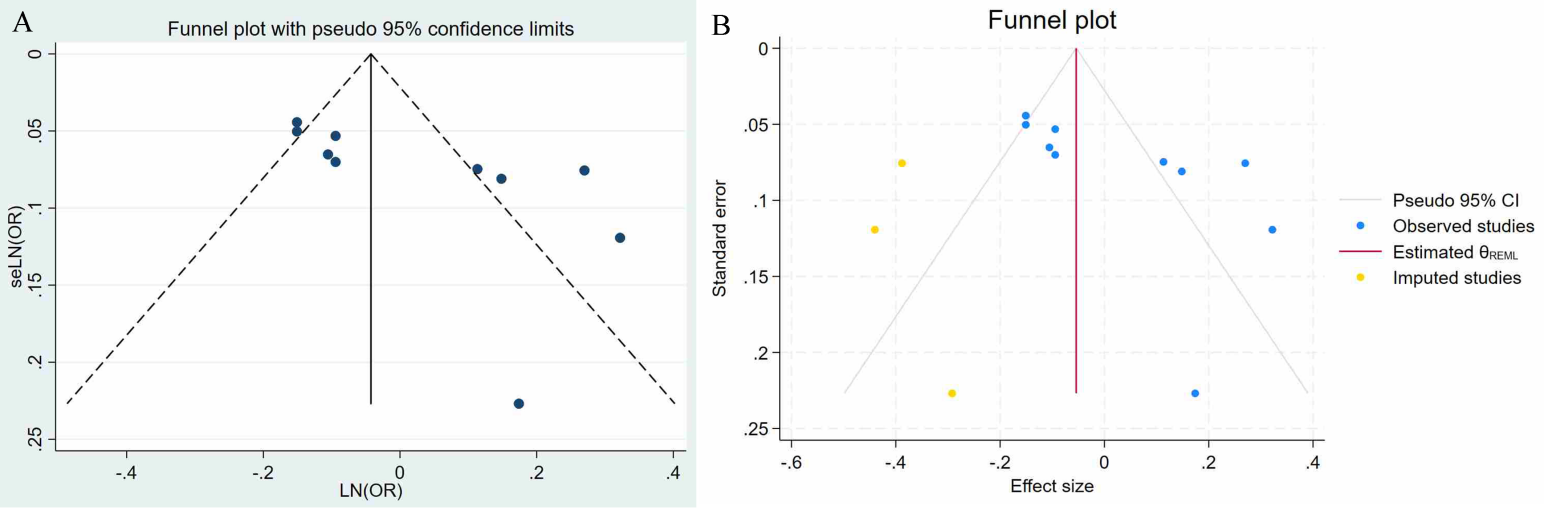
**

**Figure_S6 (**A) Funnel plot suggesting potential publication bias in studies on type 2 diabetes mellitus and liver cancer. (B) Funnel plot showing the imputed study following trim-and-fill analysis.

Abbreviations: SE,standard error; LN(OR), logarithm of the odds ratio.


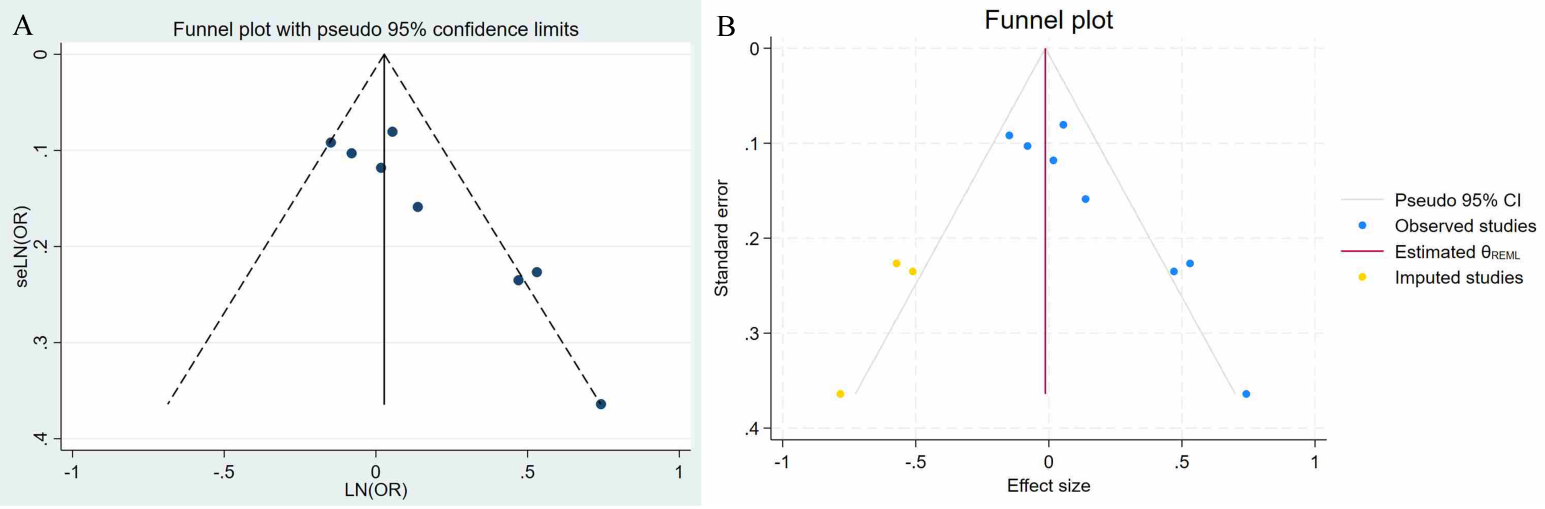


**Figure_S7 (**A) Funnel plot suggesting potential publication bias in studies on type 2 diabetes mellitus and glioblastoma. (B) Funnel plot showing the imputed study following trim-and-fill analysis.

Abbreviations: SE,standard error; LN(OR), logarithm of the odds ratio.


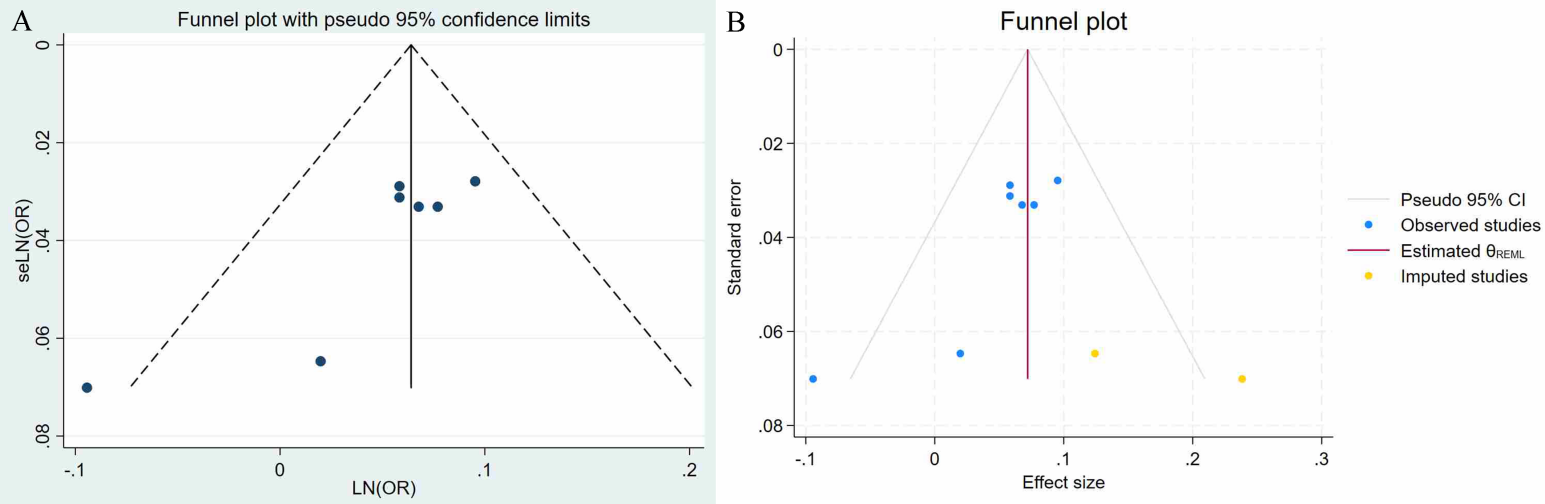


**Figure_S8 (**A) Funnel plot suggesting potential publication bias in studies on type 2 diabetes mellitus and endometrial cancer. (B) Funnel plot showing the imputed study following trim-and-fill analysis.

Abbreviations: SE,standard error; LN(OR), logarithm of the odds ratio.


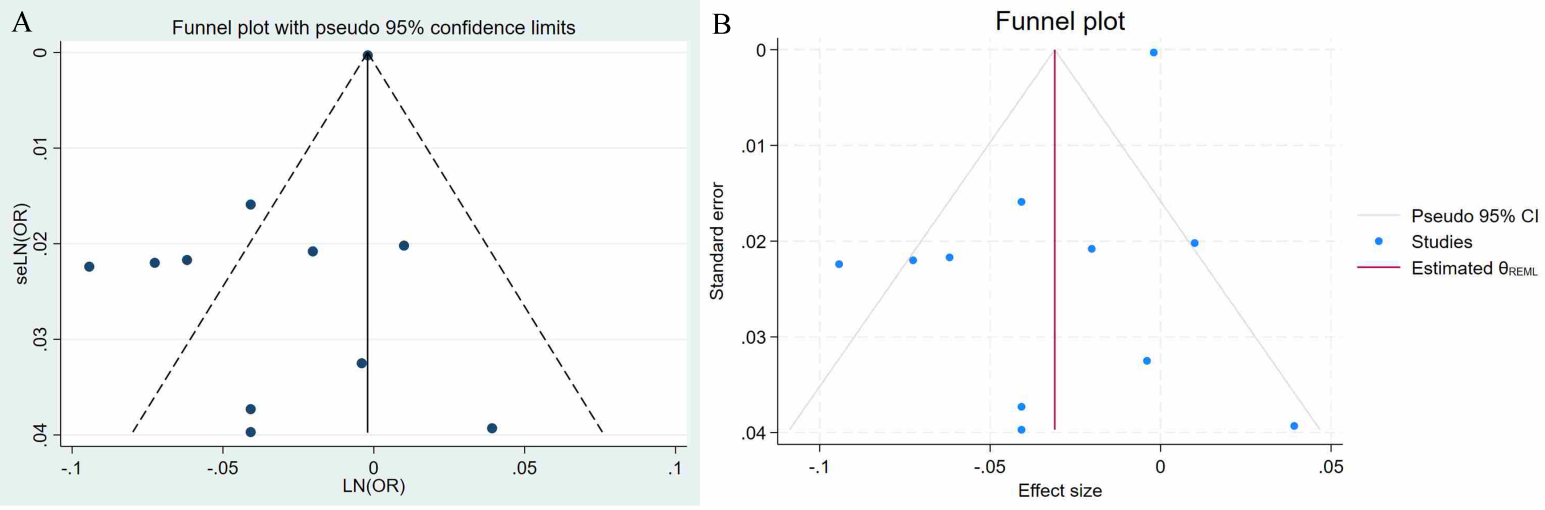


**Figure_S9 (**A) Funnel plot suggesting potential publication bias in studies on type 2 diabetes mellitus and melanoma. (B) Funnel plot showing the imputed study following trim-and-fill analysis.

Abbreviations: SE,standard error; LN(OR), logarithm of the odds ratio.

**Code S1**. R script for generating the heatmap of the association between T2DM and cancer risk (Figure 4).

setwd(‘D:/meta/T2DM_cancer’)

library(ggplot2)

library(dplyr)

library(scales)

library(readxl)

cancer_data <- read_excel(“analysis.xlsx”, sheet = “Sheet1”)

cancer_data <- cancer_data %>%

arrange(desc(OR)) %>%

mutate(Cancer = factor(Cancer, levels = Cancer))

format_2dec <- function(x) {

sprintf(“%.2f”, x)

}

p <- ggplot(cancer_data, aes(y = Cancer)) +

geom_text(aes(x = 1, label = Cancer), hjust = 0.9, size = 3.6, color = “#333333”) +

geom_tile(aes(x = 2, fill = OR), width = 0.9, height = 0.85, color = “#E6E6E6”, linewidth = 0.5) +

geom_tile(aes(x = 3, fill = OR_LL), width = 0.9, height = 0.85, color = “#E6E6E6”, linewidth = 0.5) +

geom_tile(aes(x = 4, fill = OR_UL), width = 0.9, height = 0.85, color = “#E6E6E6”, linewidth = 0.5) +

geom_text(aes(x = 2, label = format_2dec(OR)), size = 3.0, color = “#222222”, fontface = “plain”) +

geom_text(aes(x = 3, label = format_2dec(OR_LL)), size = 3.0, color = “#222222”, fontface = “plain”) +

geom_text(aes(x = 4, label = format_2dec(OR_UL)), size = 3.0, color = “#222222”, fontface = “plain”) +

scale_fill_gradientn(

colors = c(

“#000080”, “#001188”, “#003399”, “#0055AA”, “#5588BB”,

“#DDDDDD”, “#EE8877”, “#DD6655”, “#CC4433”, “#BB2211”, “#990000”

),

limits = c(0.7, 1.3),

breaks = seq(0.7, 1.3, 0.1),

name = “OR Value”,

values = rescale(c(0.7, 0.75, 0.8, 0.9, 0.95, 1.0, 1.05, 1.1, 1.2, 1.25, 1.3)),

guide = guide_colorbar(

barheight = unit(5, “cm”),

barwidth = unit(0.5, “cm”),

ticks.linewidth = 0.5,

frame.linewidth = 0.5,

title.position = “top”,

title.hjust = 0.5,

title.vjust = 1,

label.vjust = 1

)

) +

scale_x_continuous(

limits = c(0.2, 4.8),

breaks = c(1, 2, 3, 4),

labels = c(“Cancer Type”, “OR”, “OR_LL”, “OR_UL”),

expand = c(0, 0)

) +

theme_bw() +

theme(

axis.text.y = element_blank(),

axis.title.y = element_blank(),

axis.ticks = element_blank(),

panel.grid = element_blank(),

panel.border = element_blank(),

axis.text.x = element_text(size = 10, face = “bold”, color = “#333333”, margin = margin(t = 8)),

axis.title.x = element_blank(),

legend.title = element_text(size = 7, face = “bold”, color = “#333333”, margin = margin(b = 3)),

legend.text = element_text(size = 6, color = “#333333”),

legend.position = “right”,

legend.background = element_blank(),

legend.key = element_blank(),

legend.margin = margin(0, 0, 0, 0),

plot.margin = margin(10, 5, 10, 10, “mm”),

panel.background = element_rect(fill = “white”, color = NA),

plot.background = element_rect(fill = “white”, color = NA)

)

print(p)
